# Impact of solriamfetol treatment on sleep quality in Chinese patients with OSA-EDS: results of a randomized controlled trial

**DOI:** 10.3389/fmed.2026.1712097

**Published:** 2026-02-18

**Authors:** Fen Wang, Liying Deng, Liang Xie, Jiaomei Jiang, Xiang Li, Weijia Zhang, Hui Ye, Yongmin Ding

**Affiliations:** 1 Department of Neurology, The Second Affiliated Hospital of Nanchang University, Nanchang, Jiangxi, China; 2 Ignis Therapeutics (Shanghai) Limited, Shanghai, China

**Keywords:** excessive daytime sleepiness, obstructive sleep apnea, sleep parameter, sleep quality, solriamfetol, total sleep time

## Abstract

**Introduction:**

Wake-promoting agents (WPAs), such as solriamfetol, have emerged as effective treatment options for treating excessive daytime sleepiness (EDS) in patients with obstructive sleep apnea (OSA). However, the impact of solriamfetol on nocturnal sleep quality remains underexplored. This exploratory analysis of a 12-week, randomized, double-blind, placebo-controlled, multicenter, parallel-arm, phase 3 trial involving Chinese patients with OSA-EDS evaluated the effect of solriamfetol on nocturnal sleep quality.

**Methods:**

Participants were randomized (1:1) to receive solriamfetol (150 mg) or placebo once daily. The coprimary efficacy endpoints were the changes from baseline to week 12 in mean sleep latency during the Maintenance Wakefulness Test and in Epworth Sleepiness Scale scores. The exploratory endpoints included changes in participants’ polysomnography (PSG) parameters from baseline at Weeks 2, 5, and 12. These parameters were unadjusted and hypothesis-generating. The sleep quality parameters included total sleep time (TST) and wakefulness after sleep onset (WASO). Respiratory parameters included mean oxygen saturation (SaO_2_), minimum SaO_2_, apnea index, and apnea-hypopnea index (AHI).

**Results:**

Of the 357 participants screened, 201 were included in the full analysis set (FAS) (solriamfetol, *n* = 101; placebo, *n* = 100). At all-time points (Weeks 2, 5, and 12), no significant changes in TST, WASO, stage N2 sleep, and AHI were observed between the solriamfetol and placebo groups (*P* > 0.05). No significant changes were observed in N1 and N3, although significant changes were observed in N1 at Week 2 (*P* = 0.0022) and N3 at Week 5 (*P* = 0.0212).

**Conclusion:**

No clinically significant or consistent changes in PSG parameters were observed compared with placebo, indicating that solriamfetol morning administration has no significant effect on nocturnal sleep parameters.

## 1 Introduction

Obstructive sleep apnea (OSA) is one of the most common sleep-related breathing disorders characterized by recurrent episodes of pharyngeal airway collapse during sleep, leading to intermittent reductions in ventilation, impaired blood gas exchange, hypoxia, hypercapnia, and sleep fragmentation (1). These physiological disturbances diminish nocturnal sleep quality, lower oxygen saturation, and contribute to impaired daytime functioning (2, 3).

In certain pathophysiological endotypes, heightened arousal responses may further exacerbate the severity of OSA, by triggering premature awakenings that destabilize ventilatory control, leading to more frequent episodes of apneas and hypopneas (4). Patients with a low arousal threshold are particularly vulnerable (5, 6); in these individuals, arousal-induced ventilatory overshoots reduce CO_2_ levels, impair upper-airway muscle tone, and increase the likelihood of subsequent airway obstruction during sleep (5, 7).

One of the primary symptoms of OSA is excessive daytime sleepiness (EDS), which refers to the inability to remain alert and awake during waking hours (8), occurring in about 40.5–58% of the patients with OSA (9). Wake-promoting agents (WPAs) have been identified as a potential solution to alleviate EDS in patients undergoing primary treatment for OSA (10). The US Food and Drug Administration (FDA) and the European Medicines Agency (EMA) have approved several WPAs for the treatment of OSA-associated EDS in adults (11–14). However, the potential impact of WPAs on patients’ nocturnal sleep quality remains underexplored. Although some studies have shown that WPAs do not significantly affect the quality or quantity of nocturnal sleep (15, 16), clinical trials of armodafinil and modafinil have highlighted a considerable risk of insomnia (17).

Solriamfetol, a dopamine and norepinephrine reuptake inhibitor (DNRI) (18), selectively binds to and inhibits reuptake at dopamine and norepinephrine transporters without inducing the release of these neurotransmitters (19). The efficacy of solriamfetol has been demonstrated in OSA-EDS in several trials, including the Treatment of OSA and Narcolepsy Excessive Sleepiness (TONES) trials 3–5 (20–22), with a favorable safety profile, based on which it was approved for the treatment of EDS in patients with narcolepsy or OSA in the United States (13) and the European Union (23). A phase 3 clinical trial of solriamfetol involving the Chinese population was recently completed, showing significant improvement in the mean sleep latency of the Maintenance of Wakefulness Test (MWT) and mean Epworth Sleepiness Scale (ESS) scores compared with placebo during a 12-week period (24). However, analysis of the effects of solriamfetol on nocturnal sleep remains limited. In the TONES 3 study, solriamfetol reduced the total sleep time (TST) by 12.4 min, whereas wakefulness after sleep onset (WASO) and the number of awakenings increased by 6.7 min and 0.3, respectively. However, these changes were not significant compared with the placebo group (*P* > 0.05), indicating that solriamfetol does not significantly affect nocturnal sleep quality (20). Although these findings are reassuring, they also highlight the necessity of exploring the impact of solriamfetol on respiratory-related parameters in patients with OSA who may experience breathing difficulties during sleep. Evaluating these parameters could enhance understanding of the safety profile of solriamfetol and its effects on overall sleep architecture and quality.

Thus, the aim of this study was to perform an exploratory assessment of the effects of nocturnal sleep quality in participants using data from a 12-week, randomized, double-blind, placebo-controlled, multicenter, parallel-arm, phase 3 trial.

## 2 Materials and methods

### 2.1 Study design

The current exploratory analysis used data from a 12-week, randomized, double-blind, placebo-controlled, multicenter, parallel-arm, phase 3 that was conducted to evaluate the efficacy and safety of solriamfetol in patients with OSA trial,^1^ with the number of CTR20231397. The study was conducted across 26 study centers in China between July 17, 2023, and August 19, 2024. The study was approved by the Institutional Review Board/Independent Ethics Committee at each site and performed in accordance with the Declaration of Helsinki and the China National Medical Products Administration “Good Clinical Practice (GCP)” (2020) guidelines. Written informed consent was obtained from all study participants before any screening procedures. A detailed description of the study has been reported before and are summarized briefly below (24).

### 2.2 Study participants

Eligible adults aged 18–75 years, diagnosed with OSA according to the International Classification of Sleep Disorders, Third Edition (ICSD-3) criteria, had a baseline MWT mean sleep latency of < 30 min based on the average of the first 4 of a 5-trial, 40-min MWT, a baseline score of ≥ 10 on the ESS, and a usual nightly sleep time of ≥ 6 h. To ensure generalizability of the study results, participants were eligible if they met one of the following conditions: (1) currently using a primary OSA therapy at least once a week (including positive airway pressure [PAP] therapy, oral pressure therapy, oral appliances, or upper airway stimulators), (2) had undergone a surgical intervention intended to treat the underlying airway obstruction, (3) had previously used a primary OSA therapy for at least 1 month with at least 1 documented adjustment to the therapy (e.g., change in PAP pressure or mask), and (4) a small proportion of participants who have never used a primary OSA therapy and refused to use were included, to represent the current treatment status of OSA in China. The proportion of participants who met the third and fourth conditions mentioned above was maintained below 30%, ensuring that the enrolled study population was representative of patients with OSA in China.

The primary exclusion criteria related to efficacy for enrollment included the presence of clinically relevant medical, behavioral, or psychiatric disorders other than OSA that were known to contribute to EDS; use of medications that could affect the evaluation of EDS within a period before the baseline visit corresponding to at least 5 half-lives of those medications or throughout the study duration; a usual bedtime no later than 1 a.m.; an occupation requiring nighttime or variable shift work; a current or past (within the past 2 years) diagnosis of a moderate or severe substance use disorder, as defined by the Diagnostic and Statistical Manual of Mental Disorders-5 criteria; excessive caffeine use 1 week before the baseline visit or anticipated excessive use (defined caffeine use > 600 mg/d) during the study.

### 2.3 Intervention

The study consisted of 3 phases: an initial screening and baseline period of up to 60 days, followed by a 12-week treatment period and a 2-week safety follow-up. After the completion of screening and baseline assessments, eligible participants were randomly assigned in a double-blinded manner to receive either solriamfetol (150 mg) or placebo (1:1). This randomization was stratified by primary OSA treatment (adherence, non-adherence, and currently not using any primary therapy). The definitions of adherence and non-adherence were similar to a previous solriamfetol phase 3 study conducted in Western countries (20–22). Participants were considered adherent to primary therapy if they used PAP for ≥ 4 h/night on ≥ 70% of nights or had a history of surgical intervention deemed effective in treating the airway obstruction. Participants were considered non-adherent to primary therapy if their device use was at a level lower than that specified above or if treatment with a surgical intervention was deemed non-effective. The investigator accessed an internet-based central randomization system to randomly assign study participants to treatment. The randomization code was not broken or released until all study data had been collected and accepted for analysis. The study drug or placebo was taken on an empty stomach within 1 hour of waking. All study drugs were prepared in identical opaque gelatin capsules to ensure adequate double-blinding, and all study personnel were blinded to the study treatments (data on file). The first 2 weeks of the treatment phase involved dose titration. Participants started with an oral dose of 37.5 mg once daily (QD) from days 1 to 3, followed by 75 mg QD from days 4 to 7. Depending on the clinical response and tolerability, the dosing was titrated up to 150 mg QD from the first day of the second week of the treatment period. Investigators had the option to reduce the dose once during the titration phase, while maintaining participants’ maximum tolerable dose of either 75 or 150 mg QD. The stable dose was maintained from day 4 of the second week until the end of the study treatment. After completing a 12-week treatment period, the study participants entered a 2-week safety follow-up period (24, 25).

### 2.4 Polysomnography methodology

The participants were admitted to their corresponding study sites at baseline and at weeks 2, 5, and 12 for polysomnography (PSG) and MWT to be performed on the next day. Participants were admitted to the research center each PSG visit day in the morning, with the last dose of the drug administered within 1 h of waking on visit day (approximately 8:00 AM). PSG assessments were initiated between 22:00 and 23:00 on the visit day. All PSG data were interpreted by a central interpretation agency, following a standard operating procedure as per the American Academy of Sleep Medicine (AASM) 2020 standards (26), with respiratory events scored by professional sleep technicians. PSG scorers were blinded, and standardization and inter-rater checks were performed. Hypopnea was defined as a ≥ 30% reduction in nasal airflow peak signal lasting ≥ 10 s, accompanied by either a ≥ 3% decrease in blood oxygen saturation or an arousal. The AHI was calculated as the total number of apneas and hypopneas per hour of sleep (27, 28). Furthermore, scoring variables were defined as per the criteria adapted from the AASM Manual for Scoring of Sleep and Associated Events.

### 2.5 Outcomes

The co-primary efficacy endpoints were the change from baseline to week 12 in the mean sleep latency derived from the first 4 trials of a 5-trial, 40-min MWT and the change from baseline in ESS score. The key secondary endpoint was the percentage of participants at week 12 reporting any improvement in the Patient Global Impression of Change (PGI-C) score, assessed on a 7-point scale (1 = very much improved to 7 = very much worse); improvement was defined as ratings of “very much,” “much,” or “minimally” improved. The exploratory endpoints were the changes in participants’ PSG parameters (sleep quality and respiratory parameters) from baseline at weeks 2, 5, and 12. The sleep quality–related parameters included TST and WASO. Respiratory-related parameters included mean oxygen saturation (SaO_2_), minimum oxygen saturation (mini SaO_2_), apnea index (AI), and apnea-hypopnea index (AHI). TST was the duration of Rapid Eye Movement (REM) plus non-REM (NREM) (stages N1, N2, and N3) during time in bed. Arousal was defined as an abrupt shift in electroencephalogram (EEG) frequency, which could include theta, alpha, and/or frequencies > 16 Hz but not spindles. WASO was defined as wake time after persistent sleep, whereas hypopnea was defined as a peak signal excursion drop by > 30% of pre-event baseline using nasal pressure or an acceptable alternative signal lasting > 10 s.

### 2.6 Statistical analysis

A total of 204 participants were planned for enrollment, with 102 participants each assigned to the solriamfetol group and the placebo group. The sample size was determined using Student’s *t*-test, with a 2-sided significance level of 0.05. In the present study, which is a pre-specified exploratory analysis of the parent trial, multiple endpoints were involved in the PSG analysis, with no multiplicity control planned in the Statistical Analysis Plan (SAP). Moreover, all PSG parameters were exploratory, unadjusted and hypothesis-generating. Sleep quality–related parameters, including TST, N1, N2, N3, and WASO, and respiratory-related parameters, including AI, AHI, mean SaO_2_, and mini SaO_2_, were analyzed using the mixed model for repeated measures (MMRM). For the number of central apneas, a between-group comparison was conducted using the Wilcoxon rank sum test. Missing data was not imputed. MMRM model treated missing data as MAR (Missing at Random), Wilcoxon rank sum test did not treat missing data. In this study, all laboratory test results have been monitored by study physician and verified with source document by CRA, data integrity and validity have been assured. Hence, statistical analysis did not deal with potential outliers. Additional *post hoc* sensitivity analyses were performed based on participants’ baseline AHI levels, adherence and its stability status to primary OSA therapy during the baseline period. The stability of adherence was defined as participant’s baseline adherence status to primary OSA therapy remaining consistent throughout the study period. Data were presented as least squares (LS) mean or median changes from baseline, along with their standard deviations (SD) or interquartile ranges (Q1–Q3). *P*-values were reported as nominal *P*-values. For PSG parameters apart from CA and ARO, Cohen’s d was used as effect size, calculated as the between-group difference in mean change from baseline divided by the pooled standard deviation of the changes. For CA and ARO, the effect size r was computed as the *z*-value from the Wilcoxon rank-sum test divided by the square root of the sum of the sample sizes in the two groups (29). Furthermore, due to the exploratory nature of the study, the false positive rate has not been adjusted. All analyses were performed using SAS, version 9.4.

## 3 Results

### 3.1 Participant population

**FIGURE 1:**
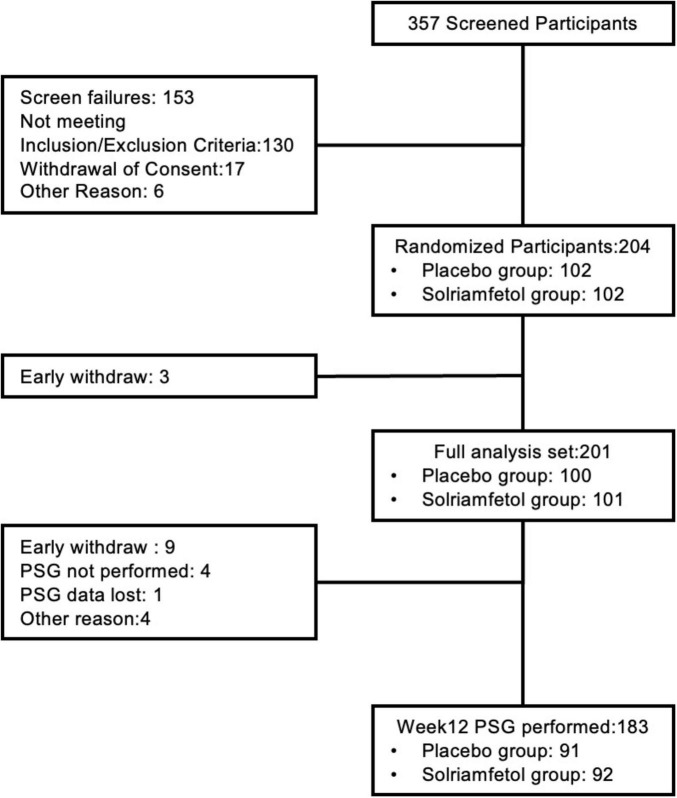
Patient disposition. Flowchart showing participant screening, randomization, and analysis sets. ESS, Epworth Sleepiness Scale; FAS, full analysis set; MWT, Maintenance of Wakefulness Test; OSA, obstructive sleep apnea; TEAE, treatment emergent adverse events.

Of the 357 participants screened, 204 were randomly assigned to receive either placebo (*n* = 102) or solriamfetol (*n* = 102), with 192 participants completing the study (solriamfetol, *n* = 96; placebo, *n* = 96). Furthermore, 201 participants were included in the FAS (solriamfetol, *n* = 101; placebo, *n* = 100) (24). In the maintenance phase, 96 participants (94.1%) received 150-mg solriamfetol, and 6 participants (5.9%) received 75-mg solriamfetol. There were 95 participants (93.1%) and 3 participants (2.9%) in the respective placebo groups. A majority of the participants were male (solriamfetol, 92.1%; placebo, 89%), with a mean age of 45.6 years (solriamfetol, 45.9 years; placebo, 45.3 years). Most participants (51.2%) were adherent to primary OSA therapy (solriamfetol, 51.5%; placebo, 51.0%), whereas 19.8% of participants in the solriamfetol group and 20.0% in the placebo group did not meet the adherence criteria. A total of 28.7% of participants in the solriamfetol group and 29.0% of participants in the placebo group were not using any primary OSA therapy. Most participants (solriamfetol, 42.5%; placebo, 42.4%) had baseline AHI < 5, whereas 19.8% and 17.2% of participants in the solriamfetol and placebo groups, respectively, had a baseline AHI between 5 and 15, respectively. Baseline 15 ≤ AHI < 30 was observed in 14.8% (solriamfetol) and 17.2% (placebo) of participants, and baseline AHI ≥ 30 was observed in 22.8% (solriamfetol) and 23.2% (placebo) of participants. At baseline, participants in the solriamfetol and placebo groups had a mean MWT sleep latency of 11.29 and 11.94 min, respectively, and a mean ESS total score of 14.8 and 14.3, respectively. A summary of the baseline demographic and clinical characteristics is presented in Table 1. The flow of participants through each stage of the study, is illustrated in Figure 1.

**TABLE 1:** Baseline demographics and patient characteristics.

Characteristics	Placebo (*N* = 100)	Solriamfetol (*N* = 101)	Total (*N* = 201)
**Age, years**			
Mean (± SD)	45.3 (± 12.61)	45.9 (± 11.83)	45.6 (± 12.20)
Median (Q1, Q3)	46.0 (35.0, 55.0)	46.0 (37.0, 54.0)	46.0 (36.0, 55.0)
**Gender, n (%)**			
Female	11 (11.0)	8 (7.9)	19 (9.5)
Male	89 (89.0)	93 (92.1)	182 (90.5)
**BMI, kg/m^2^**			
Mean (± SD)	27.32 (± 3.92)	27.74 (± 3.39)	27.53 (± 3.66)
Median (Q1, Q3)	26.65 (24.70, 29.75)	27.30 (25.80, 30.10)	27.10 (24.90, 30.00)
**Prior adherence to OSA therapy, n (%)**			
Using primary OSA therapy and meeting adherence criteria	51 (51.0)	52 (51.5)	103 (51.2)
Using primary OSA therapy but not meeting adherence criteria	20 (20.0)	20 (19.8)	40 (19.9)
Not using any primary OSA therapy	29 (29.0)	29 (28.7)	58 (28.9)
**MWT sleep latency, minutes**			
Mean (± SD)	11.940 (± 6.5757)	11.291 (± 7.1717)	11.614 (± 6.8787)
Median (Q1, Q3)	11.125 (6.50, 16.625)	8.625 (5.125, 16.125)	10.125 (5.875, 16.50)
**ESS total score**			
Mean (± SD)	14.3 (± 2.91)	14.8 (± 3.42)	14.6 (± 3.18)
Median (Q1, Q3)	14.0 (12.0, 15.5)	14.0 (12.0, 17.0)	14.0 (12.0, 16.0)
**Severity of CGIs, n (%)**			
Normal, not sick at all	1 (1.0)	2 (2.0)	3 (1.5)
Critical state	2 (2.0)	6 (5.9)	8 (4.0)
Mild	37 (37.0)	24 (23.8)	61 (30.3)
Moderate	29 (29.0)	38 (37.6)	67 (33.3)
Obvious	25 (25.0)	22 (21.8)	47 (23.4)
Severe illness	6 (6.0)	9 (8.9)	15 (7.5)

N = number of study participants in each group in the corresponding analysis population; n (%) = number and percentage of study participants meeting a specific category, percentages will be calculated based on the number of study participants in each group that is not missing; number of cases = number of non-missing study participants in a specific category in the corresponding group. BMI, body mass index; CGI, clinical global impressions; ESS, Epworth sleepiness scale; MWT, Maintenance of Wakefulness Test; OSA, obstructive sleep apnea; SD, standard deviation; Q1,Q3, first and third quartile.

### 3.2 Changes in sleep quality–related parameters

#### 3.2.1 Total sleep time and N1 to N3 sleep

**FIGURE 2:**
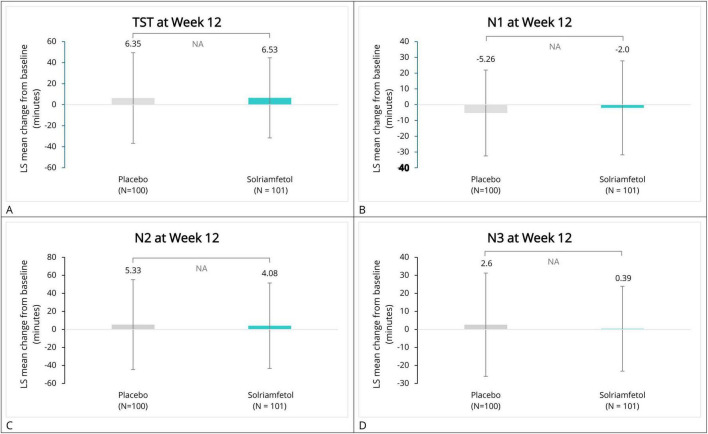
LS mean change from baseline in sleep quality–related parameters. Panels show LS mean changes (± standard error) from baseline at weeks 2, 5, and 12 for: **(A)** Total Sleep Time (TST, minutes), **(B)** Stage N1 sleep (minutes), **(C)** Stage N2 sleep (minutes), and **(D)** Stage N3 sleep (minutes). Values represent LS mean changes from baseline estimated using mixed model for repeated measures (MMRM). LS, least square; NA, not available; TST, total sleep time.

At baseline, the mean (SD) TST was similar between the groups [solriamfetol, 402.65 (42.59) min; placebo, 406.11 (39.88) min]. By week 2, a decrease in the TST was observed in both the groups. At weeks 5 and 12, an increase in the TST was observed in the solriamfetol group. However, the intergroup difference between the solriamfetol and placebo groups was not statistically significant (week 2, LS mean difference: −2.48 min [95% CI: −16.10 to 11.14]; week 5, LS mean difference: −2.81 min [95% CI: −15.58 to 9.95]; week 12, LS mean difference: −1.21 min [95% CI: −12.43 to 10.01]; all *P* > 0.05; Cohen’s *d* = 0.0045). The *P*-value of overall treatment effect for Group, Time and Group*Time was 0.6333, 0.0088 and 0.9712, respectively (Figure 2 and Table 2).

**TABLE 2:** Changes in sleep quality-related parameters from baseline to week 12 (MMRM analysis with FAS).

Variables	Placebo (*N* = 100)	Solriamfetol (*N* = 101)	Least Squares Mean Difference (95%CI) for Between-Group Comparison vs. Placebo	Intergroup P*value vs. Placebo
**TST (minutes)**				
Baseline	*N* = 99	*N* = 101		
Mean (± SD)	406.11 (39.88)	402.65 (42.59)		
Median (Q1, Q3)	409.50 (386.50, 439.00)	411.50 (368.00, 440.00)		
Week 2 change	*N* = 93	*N* = 92	−2.48 (−16.10, 11.14)	0.7199
Mean (± SD)	−4.91 (44.54)	−7.65 (52.95)		
Median (Q1, Q3)	−1.00 (−21.50, 20.00)	−2.75 (−29.75, 16.50)		
Week 5 change	*N* = 92	*N* = 96	−2.81 (−15.58, 9.95)	0.6642
Mean (± SD)	2.35 (44.09)	0.76 (45.51)		
Median (Q1, Q3)	7.25 (−17.75, 28.50)	5.75 (−18.50, 21.00)		
Week 12 change	*N* = 90	*N* = 92	−1.21 (−12.43, 10.01)	0.8316
Mean (± SD)	6.35 (43.10)	6.53 (38.12)		
Median (Q1, Q3)	9.00 (−16.00, 27.00)	2.00 (−18.75, 29.75)		
Overall treatment effect *P*-value				
Group		0.6333		
Time		0.0088		
Group*Time		0.9712		
**N1 sleep time (minutes)**				
Baseline	*N* = 99	*N* = 101		
Mean (± SD)	47.52 (29.78)	54.20 (34.48)		
Median (Q1, Q3)	41.00 (26.50, 57.00)	46.00 (33.50, 61.00)		
Week 2 change	*N* = 93	*N* = 92	9.44 (3.45, 15.43)	0.0022
Mean (± SD)	−5.81 (24.39)	0.66 (22.43)		
Median (Q1, Q3)	−6.50 (−14.50, 7.50)	0.75 (−13.00, 15.50)		
Week 5 change	*N* = 92	*N* = 96	5.75 (−0.37, 11.87)	0.0655
Mean (± SD)	−5.88 (22.67)	−3.28 (27.08)		
Median (Q1, Q3)	−3.75 (−15.50, 5.75)	−0.25 (−14.75, 10.25)		
Week 12 change	*N* = 90	*N* = 92	6.23 (−0.93, 13.38)	0.0878
Mean (SD)	−5.26 (27.25)	−2.00 (29.79)		
Median (Q1, Q3)	−2.00 (−18.00, 10.50)	−2.75 (−18.00, 9.00)		
Overall treatment effect *P*-value				
Group		0.0067		
Time		0.4284		
Group*Time		0.4570		
**N2 sleep time (minutes)**				
Baseline	*N* = 99	*N* = 101		
Mean (± SD)	233.55 (41.84)	236.97 (45.22)		
Median (Q1, Q3)	235.50 (209.00, 259.00)	238.00 (214.50, 264.00)		
Week 2 change	*N* = 93	*N* = 92	−3.87 (−15.71, 7.97)	0.5196
Mean (SD)	0.51 (43.33)	−6.99 (48.74)		
Median (Q1, Q3)	−4.50 (−24.00, 31.00)	−9.00 (−31.25, 29.00)		
Week 5 change	*N* = 92	*N* = 96	5.97 (−5.74, 17.67)	0.3159
Mean (± SD)	1.66 (44.21)	5.42 (49.72)		
Median (Q1, Q3)	2.00 (−27.00, 27.00)	2.75 (−24.25, 38.25)		
Week 12 change	*N* = 90	*N* = 92	1.53 (−10.86, 13.91)	0.8079
Mean (± SD)	5.33 (49.88)	4.08 (47.41)		
Median (Q1, Q3)	2.25 (−24.00, 34.00)	7.75 (−22.75, 26.25)		
Overall treatment effect *P*-value				
Group		0.7970		
Time		0.0673		
Group*Time		0.3762		
**N3 sleep time**				
Baseline	*N* = 99	*N* = 101		
Mean (± SD)	36.80 (32.02)	34.43 (31.05)		
Median (Q1, Q3)	28.00 (13.00, 56.00)	31.00 (4.50, 55.50)		
Week 2 change	*N* = 93	*N* = 92	−3.53 (−9.93, 2.87)	0.2785
Mean (SD)	2.53 (24.64)	0.19 (21.81)		
Median (Q1, Q3)	1.50 (−15.00, 18.50)	0.00 (−7.25, 11.00)		
Week 5 change	*N* = 92	*N* = 96	−7.79 (−14.41, −1.18)	0.0212
Mean (SD)	3.90 (25.00)	−2.96 (23.47)		
Median (Q1, Q3)	1.25 (−11.00, 16.25)	0.00 (−12.75, 9.75)		
Week 12 change	*N* = 90	*N* = 92	−3.98 (−11.27, 3.32)	0.2835
Mean (SD)	2.60 (28.66)	0.39 (23.54)		
Median (Q1, Q3)	0.25 (−14.50, 19.00)	0.00 (−12.00, 13.50)		
Overall treatment effect *P*-value				
Group		0.0671		
Time		0.7058		
Group*Time		0.3887		
**WASO (minutes)**				
Baseline	*N* = 99	*N* = 101		
Mean (± SD)	60.05 (± 35.77)	64.74 (± 40.67)		
Median (Q1, Q3)	52.00 (32.50, 82.50)	61.00 (27.50, 94.00)		
Week 2 change	*N* = 93	*N* = 92	−2.10 (−13.31, 9.11)	0.7125
Mean (± SD)	8.02 (± 37.75)	5.82 (± 43.43)		
Median (Q1, Q3)	5.50 (−15.50, 23.50)	1.25 (−14.75, 22.50)		
Week 5 change	*N* = 92	*N* = 96	1.52 (−8.12, 11.15)	0.7565
Mean (± SD)	−3.63 (± 33.91)	−3.81 (± 35.91)		
Median (Q1, Q3)	−4.75 (−20.00, 11.50)	−5.75 (−21.50, 12.25)		
Week 12 change	*N* = 90	*N* = 92	−3.58 (−13.38, 6.23)	0.4727
Mean (± SD)	−1.30 (± 36.35)	−6.22 (± 35.14)		
Median (Q1, Q3)	−4.50 (−24.00, 18.50)	−5.25 (−28.50, 14.75)		
Overall treatment effect *P-*value				
Group		0.7176		
Time		0.0012		
Group*Time		0.6603		

FAS, full analysis set; MMRM = mixed model for repeated measures; N = number of study participants in each group in the full analysis set; Number of cases = number of study participants with non-missing parameter values in the corresponding group; CI, confidence interval; SD, standard deviation; TST, total sleep time; WASO, wakefulness after sleep onset; Q1, Q3, first and third quartile. Analysis was performed using a mixed model for repeated measures (MMRM) with change from baseline in PSG parameters as the dependent variable. Fixed effects included treatment group, visit, randomization stratification factors, and treatment-by-visit interaction. Participant’s baseline value was included as a covariate. An unstructured covariance matrix was specified for all models. *Overall *P* value derived from a mixed-effects model, with change from baseline in PSG as dependent variable, treatment group by visit interaction as fixed effect, and corresponding baseline value as covariate.

For N1, N2, and N3 sleep, no significant intergroup differences between the solriamfetol and placebo groups were observed at all-time points except for N1 sleep at week 2 (LS mean difference: 9.44 [95% CI: 3.45–15.43]; *P* = 0.0022), and N3 sleep at week 5 (LS mean difference: −7.79 [95% CI: −14.41 to −1.18]; *P* = 0.0212). Cohen’s *d* values for the between-group changes were small (N1 = 0.1140; N2 = −0.0257; N3 = −0.0843). However, the overall treatment Group*Time effect of N1 and was 0.4570 and 0.3887 (Figure 2 and Table 2).

#### 3.2.2 Wakefulness after sleep onset

In comparison with the baseline (solriamfetol, 64.74 [40.67] min; placebo, 60.05 [35.77] min), WASO was initially increased by 5.82 (43.43) min at week 2 (vs. placebo, 8.02 [37.75] min) in the solriamfetol group, whereas in the following weeks, both groups experienced minimal decreases in the WASO, with reductions of 3.81 (35.91) min by week 5 in the solriamfetol group compared with 3.63 (33.91) min in the placebo group and further decreases of 6.22 (35.14 min) in the solriamfetol group versus 1.30 (36.35 min) in the placebo group. However, the differences between the two groups were only numerical and not statistically significant (week 2, LS mean difference: −2.10 min [95% CI: −13.31 to 9.11]; week 5, LS mean difference: 1.52 min [95% CI: −8.12 to 11.15]; week 12, −3.58 min [95% CI: −13.38 to 6.23]; all *P* > 0.05; Cohen’s *d* = −0.1377). Its overall treatment showed significant Time effect (Time *P* = 0.0012) but no significant Group effect (Group *P* = 0.7176) or Group*Time effect (Group*Time *P* = 0.6603) (Table 2).

### 3.3 Changes in respiratory-related parameters

#### 3.3.1 Mean oxygen saturation

**FIGURE 3:**
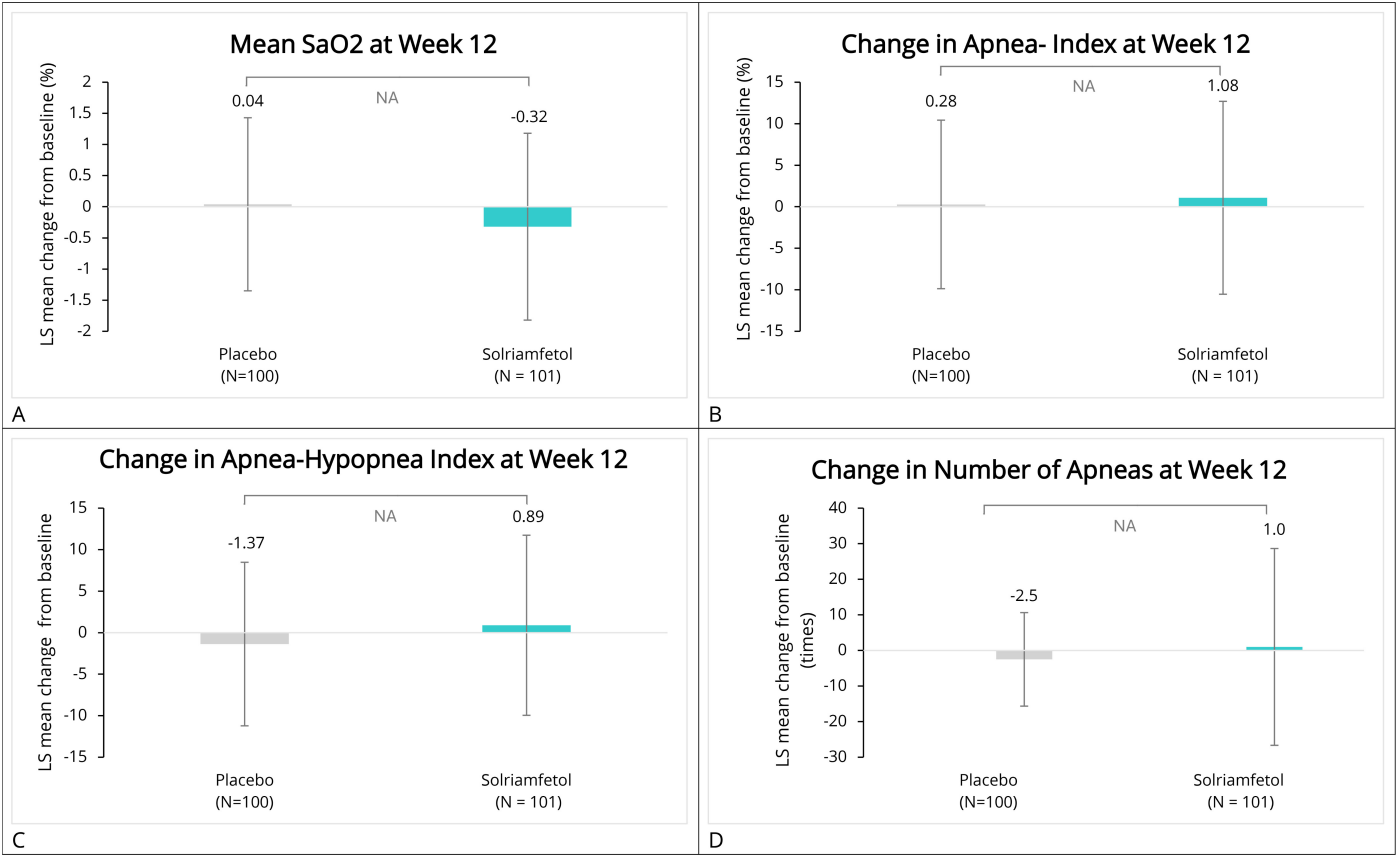
LS mean change from baseline in respiratory-related parameters. Panels show LS mean changes (± standard error) from baseline at weeks 2, 5, and 12 for: **(A)** Mean oxygen saturation (SaO2, %), **(B)** Apnea Index (AI, events/hour), **(C)** Apnea–Hypopnea Index (AHI, events/hour), and **(D)** Number of apneas (count per night). Values represent LS mean changes from baseline estimated using MMRM. LS, least squares; SaO2, oxygen saturation; AI, apnea index; AHI, apnea–hypopnea index.

When compared with the baseline (solriamfetol, 95.17% [1.74]; placebo, 95.06% [1.86]), a slight decrease in the mean SaO_2_ (by 0.14, 0.34, and 0.32%, respectively) was observed at weeks 2, 5, and 12 in participants treated with solriamfetol. Participants treated with placebo reported a slight increase in the mean SaO_2_ by 0.04, 0.14, and 0.04% at weeks 2, 5, and 12, respectively. However, these changes were not statistically significant between the two groups (week 2, LS mean difference: −0.20% [95% CI: −0.63 to 0.22]; week 5, LS mean difference: −0.40% [95% CI: −0.82 to 0.02]; week 12, −0.38% [95% CI: −0.78 to 0.02]; all *P* > 0.05; Cohen’s *d* = −0.2523). No significant overall treatment effect (Group *P* = 0.0516, Time *P* = 0.7620 and Group*Time *P* = 0.6073) was found (Figure 3A and Table 3).

**TABLE 3:** Changes in respiratory-related parameters from baseline to Week 12 (MMRM analysis with FAS).

Variables	Placebo (*N* = 100)	Solriamfetol (*N* = 101)	Least Squares Mean Difference (95%CI) vs. Placebo	Intergroup P*value vs. Placebo
**Mean oxygen saturation, n (%)**				
Baseline	*N* = 99	*N* = 101		
Mean (± SD)	95.06 (± 1.86)	95.17 (± 1.74)		
Median (Q1, Q3)	95.40 (93.90, 96.40)	95.60 (94.60, 96.40)		
Week 2 change	*N* = 93	*N* = 92	−0.20 (−0.63, 0.22)	0.3445
Mean (± SD)	0.04 (± 1.67)	−0.14 (± 1.19)		
Median (Q1, Q3)	0.10 (−0.40, 0.80)	−0.10 (−0.55, 0.50)		
Week 5 change	*N* = 92	*N* = 96	−0.40 (−0.82, 0.02)	0.0605
Mean (± SD)	0.14 (± 1.32)	−0.34 (± 1.62)		
Median (Q1, Q3)	0.00 (−0.45, 0.60)	0.00 (−0.70, 0.40)		
Week 12 change	*N* = 90	*N* = 92	−0.38 (−0.78, 0.02)	0.0637
Mean (± SD)	0.04 (± 1.39)	−0.32 (± 1.50)		
Median (Q1, Q3)	0.00 (−0.60, 0.60)	−0.15 (−0.80, 0.60)		
Overall treatment effect *P*-value				
Group		0.0516		
Time		0.7620		
Group*Time		0.6073		
**Mini SaO_2_ (%)**				
Baseline	*N* = 99	*N* = 101		
Mean (± SD)	84.02 (10.40)	84.83 (7.89)		
Median (Q1, Q3)	88.00 (79.00, 91.00)	87.00 (81.00, 90.00)		
Week 2 change	*N* = 93	*N* = 92	−0.44 (−2.03, 1.14)	0.5816
Mean (± SD)	1.03 (7.91)	0.30 (5.08)		
Median (Q1, Q3)	0.00 (−2.00, 3.00)	0.00 (−1.50, 3.00)		
Week 5 change	*N* = 92	*N* = 96	−0.72 (−2.22, 0.78)	0.3449
Mean (± SD)	1.06 (5.97)	−0.17 (5.81)		
Median (Q1, Q3)	0.00 (−2.00, 2.10)	0.00 (−4.00, 4.00)		
Week 12 change	*N* = 90	*N* = 92	−0.74 (−2.43, 0.96)	0.3922
Mean (± SD)	0.57 (7.78)	−0.61 (5.30)		
Median (Q1, Q3)	0.00 (−3.00, 3.00)	0.00 (−3.00, 2.50)		
Overall treatment effect *P*-value				
Group		0.3334		
Time		0.3455		
Group*Time		0.9118		
**AI (times/hour)**				
Baseline	*N* = 99	*N* = 101		
Mean (± SD)	6.66 (12.32)	8.32 (16.16)		
Median (Q1, Q3)	1.10 (0.10, 8.50)	1.20 (0.20, 8.40)		
Week 2 change	*N* = 93	*N* = 92	0.92 (−1.73, 3.56)	0.4965
Mean (± SD)	−0.91 (13.01)	−0.51 (7.81)		
Median (Q1, Q3)	−0.10 (−2.30, 0.70)	0.00 (−0.85, 1.00)		
Week 5 change	*N* = 92	*N* = 96	2.06 (−0.59, 4.70)	0.1266
Mean (± SD)	−0.84 (9.49)	0.68 (10.18)		
Median (Q1, Q3)	0.00 (−0.95, 0.85)	0.00 (−1.15, 1.40)		
Week 12 change	*N* = 90	*N* = 92	1.43 (−1.50, 4.35)	0.3368
Mean (± SD)	0.28 (10.15)	1.08 (11.62)		
Median (Q1, Q3)	0.00 (−1.30, 1.60)	0.00 (−0.90, 1.50)		
Overall treatment effect *P*-value				
Group		0.1716		
Time		0.1613		
Group*Time		0.6724		
**AHI (times/hour)**				
Baseline	*N* = 99	*N* = 101		
Mean (± SD)	17.58 (21.24)	18.55 (23.59)		
Median (Q1, Q3)	7.80 (1.70, 26.20)	6.60 (1.60, 27.60)		
Week 2 change	*N* = 93	*N* = 92	−1.19 (−4.97, 2.59)	0.5346
Mean (± SD)	1.11 (15.75)	−0.16 (10.14)		
Median (Q1, Q3)	−0.60 (−4.40, 2.00)	−0.30 (−2.25, 1.55)		
Week 5 change	*N* = 92	*N* = 96	2.53 (−0.31, 5.36)	0.0808
Mean (± SD)	−1.76 (10.42)	0.67 (9.77)		
Median (Q1, Q3)	−0.30 (−5.05, 2.35)	−0.10 (−2.45, 3.15)		
Week 12 change	*N* = 90	*N* = 92	2.50 (−0.46, 5.45)	0.0968
Mean (± SD)	−1.37 (9.85)	0.89 (10.85)		
Median (Q1, Q3)	−0.35 (−4.50, 2.10)	−0.30 (−2.40, 1.90)		
Overall treatment effect *P*-value				
Group		0.3024		
Time		0.7025		
Group*Time		0.1085		
**Number of apneas (times)**				
Baseline	*N* = 99	*N* = 101		
Mean (± SD)	7.1 (± 16.32)	4.5 (± 18.81)		
Median (Q1, Q3)	1.0 (0.0, 6.0)	1.0 (0.0, 3.0)		
Week 2 change	*N* = 93	*N* = 92		0.6488
Mean (± SD)	−2.0 (± 15.71)	−1.4 (± 17.65)		
Median (Q1, Q3)	0.0 (−2.0, 2.0)	0.0 (−1.0, 1.0)		
Week 5 change	*N* = 92	*N* = 96		0.7743
Mean (± SD)	−1.4 (± 11.66)	−0.9 (± 18.03)		
Median (Q1, Q3)	0.0 (−2.0, 1.0)	0.0 (−2.0, 1.0)		
Week 12 change	*N* = 90	*N* = 92		0.8745
Mean (± SD)	−2.5 (± 13.14)	1.0 (± 27.66)		
Median (Q1, Q3)	0.0 (−3.0, 1.0)	0.0 (−1.0, 0.5)		

FAS, full analysis set; MMRM = mixed model for repeated measures; N = number of study participants in each group in the full analysis set; Number of cases = number of study participants with non-missing parameter values in the corresponding group; CI, confidence interval; AHI, apnea-hypopnea index; AI, apnea index; Mini SaO_2_, minimum oxygen saturation; SD, standard deviation. Analysis was performed using a mixed model for repeated measures (MMRM) with change from baseline in PSG parameters as the dependent variable. Fixed effects included treatment group, visit, randomization stratification factors, and treatment-by-visit interaction. Participant’s baseline value was included as a covariate. An unstructured covariance matrix was specified for all models.

#### 3.3.2 Minimum oxygen saturation

At baseline, the mini SaO_2_ was similar for participants treated with solriamfetol (84.83% [7.89]); placebo (84.02% [10.4]). When compared with the baseline, in participants treated with solriamfetol, a slight numerical increase in the mini SaO_2_ by 0.30% was observed at week 2 and a slight decrease in mini SaO_2_ by 0.17% was observed at week 5 and 0.61% at week 12. Participants treated with placebo reported a minimal numerical increase by 1.03, 1.06, and 0.57% at weeks 2, 5, and 12, respectively, with insignificant intergroup differences (week 2, LS mean difference: −0.44% [95% CI: −2.03 to 1.14]; week 5, LS mean difference: −0.72% [95% CI: −2.22 to 0.78]; week 12, −0.74% [95% CI: −2.43 to 0.96]; all *P* > 0.05; Cohen’s *d* = −0.1770). No significant overall treatment effect (Group *P* = 0.3334, Time *P* = 0.3455 and Group*Time *P* = 0.9118) was found (Table 3).

#### 3.3.3 Apnea index

At week 2, when compared with the baseline, solriamfetol and placebo minimally decreased apnea index (AI) by 0.51 (7.81) and 0.91 (13.01), respectively. Solriamfetol slightly increased AI by 0.68 (10.18) at week 5 and by 1.08 (11.62) at week 12, whereas those on placebo initially reported a slight decrease of 0.84 (9.49) at week 5 followed by a slight increase of 0.28 (10.15) at week 12. At week 12, when compared with the baseline, both solriamfetol and placebo slightly increased AI. However, the differences between the two groups were not statistically significant (week 2, LS mean difference: 0.92 [95% CI: −1.73 to 3.56]; week 5, LS mean difference: 2.06 [95% CI: −0.59 to 4.70]; week 12, LS mean difference: 1.43 [95% CI: −1.50 to 4.35]; all *P* > 0.05; Cohen’s *d* = 0.0739). No significant overall treatment effect (Group *P* = 0.1716, Time *P* = 0.1613 and Group*Time *P* = 0.6724) was found (Figure 3B and Table 3).

#### 3.3.4 Apnea-hypopnea index

After an initial decrease by 0.16 (10.14) at week 2, solriamfetol slightly increased AHI by 0.67 (9.77) and 0.89 (10.85) at weeks 5 and 12, respectively, compared with the baseline. Conversely, placebo initially increased AHI by 1.11 (15.75), followed by a decrease in the AHI by 1.76 (10.42) and 1.37 (9.85) at weeks 5 and 12, respectively. Moreover, the intergroup differences were not statistically significant (week 2, the LS mean difference: −1.19 [95% CI: −4.97 to 2.59]; week 5, the LS mean difference: 2.53 [95% CI: −0.31 to 5.36); week 12, the LS mean difference: 2.50 [95% CI: −0.46 to 5.45]; all *P* > 0.05; Cohen’s *d* = 0.2184]. No significant overall treatment effect (Group *P* = 0.3024, Time *P* = 0.7025 and Group*Time *P* = 0.1085) was found (Figure 3C and Table 3).

#### 3.3.5 Number of apneas

At baseline, the mean (SD) number of apneas per night was 4.5 (18.81) and 7.1 (16.32) for the participants in the solriamfetol group and the placebo group, respectively. At week 2, the number of apneas was slightly decreased by 1.4 (17.65) and 2.0 (15.71), respectively, for the participants in the solriamfetol and placebo groups. A similar trend was observed at week 5 with a decrease by 0.9 (18.03) followed by an increase by 1.0 (27.66) at week 12 for the participants in the solriamfetol group. However, in the placebo group, the decrease was coherent from 1.4 (11.66) to 2.5 (13.14) at weeks 5 and 12, respectively. The intergroup differences were only numerical and not statistically significant, all *P* > 0.05; effect size *r* = −0.0117 (Figure 3D and Table 3).

### 3.4 Sensitivity analyses

Sensitivity analysis of the PSG parameters stratified by primary therapy adherence showed consistent results with the overall analysis. No statistically significant intergroup difference between the solriamfetol and placebo groups for TST, N2 sleep, WASO, mean SaO_2_, mini SaO_2_, AI, AHI and number of apneas with all *P* > 0.05 at week 12, except for N1 sleep (LS mean difference: 8.04 [95% CI: 0.95–15.13]; *P* = 0.0267), N3 sleep (LS mean difference: −11.67 [95% CI: −22.65, −0.69]; *P* = 0.0375), AHI (LS mean difference: 3.12 [95% CI: 0.02–6.22]; *P* = 0.0485) in the adherent group, and mean SaO_2_ (LS mean difference: −1.17 [95% CI: −2.06 to −0.27]; *P* = 0.0126) in the non-adherent group (Table 4).

**TABLE 4:** Analysis of PSG changes from baseline – stratified by primary therapy adherence.

Variables	Placebo			Solriamfetol		
	Adherent to primary therapy (*N* = 51)	Non-adherent to primary therapy (*N* = 20)	No primary therapy (*N* = 29)	Adherent to primary therapy (*N* = 52)	Non-adherent to primary therapy (*N* = 20)	No primary therapy (*N* = 29)
**TST (minutes)**						
Baseline						
Number	51	19	29	52	20	29
Mean (± SD)	407.21 (38.804)	402.76 (41.907)	406.38 (41.705)	405.54 (39.399)	401.48 (37.657)	398.29 (51.502)
Median (Q1, Q3)	410.50 (383.00, 439.00)	405.00 (378.50, 440.00)	409.00 (391.00, 433.00)	409.75 (370.50, 439.25)	408.00 (370.50, 434.00)	412.00 (361.50, 443.50)
Week 12 (change from baseline)						
Number	45	18	27	50	17	25
Mean (± SD)	7.27 (32.203)	11.47 (54.406)	1.41 (51.218)	3.43 (39.470)	1.82 (42.319)	15.94 (31.736)
Median (Q1, Q3)	8.50 (−15.50, 20.00)	16.50 (0.50, 36.00)	4.50 (−29.00, 43.00)	2.00 (−24.50, 29.00)	−7.50 (−20.00, 43.50)	12.50 (−8.00, 37.50)
Least Squares Mean Difference (95%CI) vs Placebo				−4.63 (−18.18, 8.92)	−8.23 (−41.46, 25.00)	9.52 (−12.69, 31.73)
*P*-value				0.4991	0.6180	0.3932
**N1 sleep time (minutes)**						
Baseline						
Number	51	19	29	52	20	29
Mean (± SD)	42.28 (27.395)	45.87 (33.466)	57.79 (29.702)	42.64 (21.719)	50.73 (40.330)	77.33 (38.360)
Median (Q1, Q3)	37.50 (23.00, 52.50)	38.50 (26.00, 52.00)	54.00 (44.50, 72.00)	38.25 (28.25, 52.00)	45.75 (31.75, 51.75)	67.50 (52.00, 100.50)
Week 12 (change from baseline)						
Number	45	18	27	50	17	25
Mean (± SD)	−8.30 (25.878)	−8.14 (25.035)	1.74 (30.418)	0.04 (24.167)	−7.56 (22.909)	−2.30 (42.330)
Median (Q1, Q3)	−2.00 (−21.00, 6.50)	−5.50 (−14.00, 6.50)	5.00 (−21.00, 17.00)	−0.25 (−11.50, 10.00)	−6.00 (−13.50, 7.50)	−15.00 (−25.00, 7.50)
Least squares mean difference (95%CI) vs. placebo				8.04 (0.95, 15.13)	2.84 (−8.12, 13.79)	2.71 (−17.13, 22.56)
*P-*value				0.0267	0.6022	0.7852
**N2 sleep time (minutes)**						
Baseline						
Number	51	19	29	52	20	29
Mean (± SD)	232.26 (38.393)	240.84 (51.782)	231.02 (41.553)	242.96 (46.825)	245.08 (39.690)	220.64 (43.004)
Median (Q1, Q3)	234.50 (209.00, 251.00)	249.50 (204.50, 279.00)	233.50 (216.50, 254.00)	245.75 (221.75, 265.75)	250.00 (219.25, 272.50)	226.50 (196.00, 242.50)
Week 12 (changed from baseline)						
Number	45	18	27	50	17	25
Mean (± SD)	5.92 (51.896)	9.06 (43.387)	1.87 (52.005)	1.15 (47.429)	2.56 (53.683)	10.98 (44.048)
Median (Q1, Q3)	1.00 (−24.00, 34.00)	6.00 (−20.00, 35.00)	9.00 (−34.00, 30.00)	7.25 (−25.00, 21.50)	20.50 (−33.00, 31.50)	10.50 (−15.00, 26.00)
Least squares mean difference (95%CI) vs. Placebo				4.78 (−11.94, 21.50)	−3.95 (−32.50, 24.59)	5.07 (−20.61, 30.74)
*P*-value				0.5715	0.7802	0.6936
**N3 sleep time (minutes)**						
Baseline						
Number	51	19	29	52	20	29
Mean (± SD)	40.34 (31.332)	32.32 (23.829)	33.50 (37.694)	37.79 (30.433)	34.78 (33.312)	28.17 (30.690)
Median (Q1, Q3)	37.50 (16.50, 60.00)	27.50 (8.00, 53.00)	24.50 (7.50, 42.50)	39.75 (7.50, 55.25)	30.25 (3.25, 66.50)	19.50 (0.00, 50.00)
Week 12 (changed from baseline)						
Number	45	18	27	50	17	25
Mean (± SD)	7.31 (32.061)	0.33 (21.789)	−3.74 (26.040)	−2.16 (25.546)	0.76 (23.001)	5.24 (19.452)
Median (Q1, Q3)	5.00 (−8.00, 21.50)	0.00 (−14.50, 11.50)	−2.00 (−16.50, 19.00)	1.00 (−20.50, 12.00)	3.50 (−4.50, 8.00)	0.00 (−5.00, 15.00)
Least Squares mean difference (95%CI) vs. Placebo				−11.67 (−22.65, −0.69)	1.49 (−14.17, 17.15)	6.28 (−5.81, 18.37)
*P*-value				0.0375	0.8474	0.3019
**WASO (minutes)**						
Baseline						
Number	51	19	29	52	20	29
Mean (± SD)	61.19 (36.177)	58.26 (35.168)	59.22 (36.603)	62.99 (40.723)	67.48 (38.628)	66.00 (43.150)
Median (Q1, Q3)	53.50 (32.00, 83.50)	49.00 (28.50, 89.50)	52.00 (41.00, 69.50)	57.75 (27.50, 93.25)	62.75 (28.50, 100.50)	65.00 (23.00, 98.50)
Week 12 (changed from baseline)						
Number	45	18	27	50	17	25
Mean (± SD)	−4.52 (29.088)	−0.33 (44.639)	3.43 (41.775)	−3.18 (36.179)	−6.41 (42.749)	−12.18 (27.087)
Median (Q1, Q3)	−4.50 (−23.00, 14.00)	−5.50 (−31.00, 23.50)	−7.00 (−27.50, 36.00)	−4.50 (−19.50, 18.00)	10.00 (−46.00, 18.50)	−6.00 (−30.50, 0.00)
Least squares mean difference (95%CI) vs. Placebo				1.97 (−9.79, 13.74)	−6.16 (−36.02, 23.70)	−13.17 (−32.12, 5.78)
*P*-value				0.7400	0.6776	0.1690
**AHI (times/hour)**						
Baseline						
Number	51	19	29	52	20	29
Mean (± SD)	8.56 (14.338)	17.93 (20.963)	33.21 (22.968)	6.03 (12.926)	20.23 (24.191)	39.83 (22.864)
Median (Q1, Q3)	2.50 (0.80, 9.50)	8.30 (2.40, 25.60)	30.90 (19.70, 43.40)	2.40 (0.80, 5.40)	12.95 (1.75, 27.55)	40.50 (20.40, 52.30)
Week 12 (changed from baseline)						
Number	45	18	27	50	17	25
Mean (± SD)	−2.02 (8.408)	−2.29 (11.124)	0.33 (11.281)	1.58 (8.254)	−1.34 (9.940)	1.05 (15.379)
Median (Q1, Q3)	−0.10 (−3.50, 1.40)	−1.65 (−5.90, 0.10)	1.20 (−4.90, 5.10)	−0.05 (−1.00, 0.90)	−0.90 (−2.90, 0.70)	−0.20 (−7.90, 12.80)
Least squares mean difference (95%CI) vs. placebo				3.12 (0.02, 6.22)	0.65 (−6.58, 7.87)	2.09 (−5.25, 9.42)
*P*-value				0.0485	0.8570	0.5710
**AI (times/hour)**						
Baseline						
Number	51	19	29	52	20	29
Mean (± SD)	2.06 (3.962)	8.71 (19.774)	13.39 (12.782)	2.90 (10.660)	8.00 (15.462)	18.26 (20.156)
Median (Q1, Q3)	0.40 (0.10, 1.50)	1.50 (0.30, 5.70)	11.20 (1.30, 20.80)	0.40 (0.00, 1.25)	2.30 (0.80, 8.50)	9.30 (2.10, 29.70)
Week 12 (changed from baseline)						
Number	45	18	27	50	17	25
Mean (± SD)	0.17 (3.773)	−2.44 (15.640)	2.26 (12.623)	0.77 (8.874)	−1.72 (4.396)	3.61 (18.047)
Median (Q1, Q3)	0.00 (−0.80, 0.50)	−0.90 (−2.40, 0.40)	2.50 (−5.00, 9.30)	0.00 (−0.30, 0.50)	−0.80 (−2.90, 0.00)	1.50 (−6.40, 10.60)
Least squares mean difference (95%CI) vs. Placebo				0.89 (−1.58, 3.37)	0.77 (−5.49, 7.03)	3.17 (−5.27, 11.61)
*P*-value				0.4757	0.8037	0.4541
**Mean SaO_2_, n (%)**						
Baseline						
Number	51	19	29	52	20	29
Mean (± SD)	95.35 (1.898)	94.84 (1.662)	94.69 (1.873)	95.36 (1.818)	95.54 (1.508)	94.59 (1.667)
Median (Q1, Q3)	95.80 (94.10, 96.80)	95.10 (93.90, 96.00)	95.10 (93.50, 96.00)	95.80 (94.60, 96.70)	96.10 (95.05, 96.40)	94.80 (93.80, 95.90)
Week 12 (changed from baseline)						
Number	45	18	27	50	17	25
Mean (± SD)	−0.00 (1.380)	0.87 (1.489)	−0.44 (1.092)	−0.20 (1.445)	−0.47 (1.182)	−0.48 (1.789)
Median (Q1, Q3)	−0.10 (−0.50, 0.70)	0.55 (0.00, 1.20)	−0.20 (−1.50, 0.30)	0.05 (−0.70, 0.50)	−0.60 (−1.20, 0.20)	−0.50 (−0.90, 0.70)
Least squares mean difference (95%CI) vs. Placebo				−0.24 (−0.77, 0.30)	−1.17 (−2.06, −0.27)	−0.08 (−0.91, 0.75)
*P*-value				0.3859	0.0126	0.8448
**Mini SaO_2_, n (%)**						
Baseline						
Number	51	19	29	52	20	29
Mean (± SD)	87.51 (8.169)	81.95 (11.083)	79.24 (11.479)	87.48 (7.163)	84.95 (5.978)	80.00 (8.194)
Median (Q1, Q3)	91.00 (86.00, 92.000	87.00 (77.00, 90.00)	81.00 (73.00, 88.00)	89.50 (86.50, 92.00)	85.00 (82.00, 89.50)	81.00 (76.00, 83.00)
Week 12 (changed from baseline)						
Number	45	18	27	50	17	25
Mean (± SD)	0.44 (6.066)	4.83 (11.247)	−2.07 (6.492)	−0.20 (4.772)	−0.82 (4.694)	−1.28 (6.662)
Median (Q1, Q3)	−1.00 (−2.00, 2.00)	1.50 (−1.00, 5.00)	−3.00 (−5.00, 2.00)	0.00 (−2.00, 2.00)	−1.00 (−3.00, 2.00)	0.00 (−4.00, 4.00)
Least squares mean difference (95%CI) vs. placebo				−0.81 (−2.85, 1.23)	−3.59 (−8.05, 0.87)	1.35 (−2.05, 4.75)
*P*-value				0.4299	0.1108	0.4280

The data presents the number of participants with non-missing value at visit; CI, confidence interval. AHI, apnea-hypopnea index; AI, apnea index; Mini SaO_2_, minimum oxygen saturation; SD, standard deviation. TST, total sleep time; WASO, wakefulness after sleep onset; Q1, Q3, first and third quartile.

Another sensitivity analysis of the PSG parameters in participants whose adherence status to primary OSA therapy remain stable revealed consistent results with the overall analysis. No statistically significant intergroup difference between the solriamfetol and placebo groups for TST, N1 sleep, N2 sleep, N3 sleep, WASO, mini SaO_2_, AI, AHI and number of apneas with all *P* > 0.05 at week 12, while mean SaO_2_ at week 12 (LS mean difference: −0.46 [95% CI: −0.90 to −0.02]; *P* = 0.0427) (Supplementary Tables 1, 2).

Sensitivity analysis of the PSG parameters stratified by baseline AHI showed consistent results with the overall analysis. No statistically significant intergroup difference between the solriamfetol and placebo groups for TST, N2 sleep, WASO, mean SaO_2_, mini SaO_2_, AI, AHI and number of apneas with all *P* > 0.05 at week 12, except for N3 sleep (LS mean difference: −13.57 [95% CI: −25.49 to −1.64]; *P* = 0.0263) in the AHI < 5 group, and AHI (LS mean difference: 6.26 [95% CI: 0.67–11.86]; *P* = 0.0294), mean SaO_2_ (LS mean difference: −1.05 [95% CI: −1.96 to −0.14]; *P* = 0.0251), mini SaO_2_ (LS mean difference: −5.04 [95% CI: −9.36 to −0.73]; *P* = 0.0235) in the 5 ≤ AHI < 15 group (Supplementary Tables 3, 4).

### 3.5 Safety

Throughout the study, only 3 cases of insomnia adverse events (AEs) were reported, all of which occurred in the placebo group.

## 4 Discussion

In this study involving Chinese patients with OSA-EDS, treatment with solriamfetol showed no significant effects on nocturnal sleep or wake parameters. Importantly, this lack of observed impact on nighttime sleep reflects the timing of morning administration, rather than indicating an absence of any potential drug influence on nocturnal sleep. This clinical trial monitored nocturnal respiratory indicators in participants with varying adherence to treatment, including those who declined basic OSA treatment. At week 12, treatment with solriamfetol resulted in a slight increase in TST, AI, and AHI, while reducing WASO, mean SaO_2_, and mini SaO_2_ compared with placebo. However, these effects were not statistically significant compared with baseline and did not differ from the placebo group.

There were no differences in this study except for individual time points, such as N1 at week 2 (*P* = 0.0022) and N3 at week 5 (*P* = 0.0212). However, the differences were exploratory, not corrected for multiplicity, and should be considered hypothesis-generating, and may represent false positives. Besides, the distribution of sleep stages showed an inconsistent pattern associated with solriamfetol. The absence of a clear trend suggests that solriamfetol may not significantly influence the typical progression through these stages in a clinically meaningful manner. Similarly, WASO, which measures the total minutes spent awake after the first sleep onset, serves as an indicator of sleep fragmentation. Consequently, an increase in the WASO correlates with a decrease in the sleep efficiency (30). As WASO is an indicator of sleep fragmentation, and patients with OSA often suppress awakening because of apneic episodes, it is essential to study this as an indicator to better understand disruptions in their sleep patterns (31, 32). This parameter should be studied especially when using WPAs to ensure that patients with OSA receive appropriate treatment and that WPAs do not inadvertently mask or exacerbate the underlying condition (33). In the present study, there was no significant difference in WASO at all-time points (*P* > 0.05). These findings suggest that solriamfetol may not significantly change the restorative quality of sleep.

The AHI, AI, and SaO_2_ levels of patients with OSA need to be regularly monitored because these indicators provide crucial insights into the severity of their condition, its effects on overall health, and the potential for serious complications. Collectively, AHI and AI are considered predictors of clinically significant outcomes related to OSA, including patient-reported outcomes of daytime sleepiness and quality of life, motor vehicle and industrial accidents, various cardiovascular diseases, and mortality (34). Although WPAs can be beneficial, it is important to understand that they typically do not restore nighttime sleep to a level of alertness comparable with daytime wakefulness, nor do they address the health issues associated with disrupted sleep. By merely masking sleepiness without addressing the root problem, WPAs may allow untreated OSA to persist, thereby increasing the risk of cardiovascular and metabolic diseases and further disrupting sleep architecture. Furthermore, WPAs may lead to irregular sleep patterns or reduced sleep efficiency in patients with OSA, potentially worsening sleep quality (35). This highlights the importance of close monitoring of AHI, AI, and SaO_2_ to ensure optimal management of OSA-EDS. Participants were stratified according to their AHI into 4 subgroups: AHI < 5 times/h, AHI from 5 to 15 times/h, AHI from 15 to 30 times/h, and AHI ≥ 30 times/h. Nearly half of the participants in the present study were already using continuous positive airway pressure (CPAP), and hence, the severity of the disease for these participants could not be ascertained. Furthermore, changes in respiratory parameters were minimal when compared with the placebo group, which indicates that solriamfetol does not exacerbate sleep disordered breathing, limiting its clinical implications. All calculated effect size values at week 12 in this study fall within the very small to small range, indicating that the observed differences between solriamfetol and placebo are of negligible practical magnitude and unlikely to be clinically meaningful. Taken together, these small effect sizes (Cohen’s d or *r* < 0.3) and differences well below literature-suggested clinical thresholds collectively confirm the absence of any clinically meaningful impact of solriamfetol on nocturnal sleep parameters (36–38).

Our PSG results resonated with the adverse event (AE) profile of solriamfetol clinical trials. The most common AE was dizziness, and upper respiratory infection was observed in both the placebo and solriamfetol groups. These results were also consistent with those from earlier TONES trials (20, 22). Since the present manuscript focuses on the analysis of nighttime sleep, we have reported sleep-related AEs. Only 3 cases of insomnia were reported, all in the placebo group, with no insomnia occurring in the solriamfetol group. These findings are consistent with the TONES 3 study. In the TONES3 study, insomnia was reported 3 (2.6%) participants in solriamfetol 150 mg group and 0 in 75 mg. There were also no reports of AE suggestive of euphoria, consistent with previous studies (39).

Our results were consistent with PSG findings from other WPAs. A 12-week study involving participants with OSA reported similar results, with no significant differences observed for modafinil compared with the baseline (WASO, 50 min vs. 47 min; arousal index, 4.5 vs. 3) (15). Similarly, in a 12-week trial, armodafinil demonstrated no clinically significant differences in PSG parameters compared with placebo (WASO, 71.5 min vs. 70.5 min) (16). Modafinil also demonstrated similar findings versus placebo at week 12, with an AHI of 4.3 versus 4.2 (15). A 4-week study also reported no significant differences in AHI between modafinil and placebo compared with the baseline (2.6 vs. 2.6) (40). Similar results of having no significant changes in sleep architecture or sleep quality were reported by participants treated with pitolisant (41).

One possible explanation for the limited effect of solriamfetol on nocturnal sleep is the that solriamfetol has a half-life of approximately 6–7.6 h (42). This duration is shorter compared with other WPAs, such as modafinil and armodafinil (42), that typically have longer half-lives ranging from 9 to 14 h. The shorter half-life of solriamfetol may limit its influence on sleep, because its effects are likely to diminish before the onset of nocturnal sleep, thereby reducing its potential to disrupt sleep patterns. Therefore, solriamfetol may serve as a new choice of WPA. However, it should also be noted that the purpose of this study was not to promote casual treatment of OSA, and CPAP remains the first-line treatment for OSA.

Although numerical changes were observed at week 12 compared with the baseline, including a slight increase in the AHI, a reduction in the mean SaO_2_, and reduced N1 sleep duration, these differences were not statistically significant. As such, they reflect natural variability rather than a drug-related effect. Notably, the absence of statistical significance suggests that these changes should be interpreted with caution. Given the relatively short half-life of solriamfetol and the long drug-to-PSG interval, plasma concentrations at the time of measurement were estimated to be low, further limiting its direct pharmacologic influence on sleep parameters. It is important to note that the study was designed for morning administration of the treatment and the data were collected from clinical research settings rather than real-world scenarios. This distinction highlights the importance of reminding patients in routine clinical practice to maintain adequate nocturnal sleep duration, as this may significantly impact treatment outcomes and overall efficacy. Future research should involve extended follow-up periods and comparisons with existing OSA treatments in China to address these gaps. It should also be noted that the purpose of this study was not to promote casual treatment of OSA. Instead, the study was designed to reflect real-world clinical practice and assess the efficacy and safety of solriamfetol in patients with OSA-EDS across varying baseline conditions. Notably, at baseline, the mean sleep duration of participants receiving solriamfetol and placebo was 402.65 (42.59) min and 406.11 (39.88) min, respectively. This indicates that insufficient sleep duration was not present in the study population. In addition, the inclusion of a non-compliant population did not affect the study results, underscoring that the use of solriamfetol does not change the nighttime sleep quality of patients with OSA.

In addition, we specifically conducted 3 sensitivity analyses: stratified by primary treatment compliance, by the stability of the primary OSA therapy treatment stratified by baseline AHI levels. The results of all sensitivity analyses were consistent with the overall study findings—solriamfetol had no significant impact on sleep parameters. However, differences were observed in a few individual endpoints. For example, a difference in N3 sleep was observed in the AHI < 5 subgroup (*P* = 0.021). These discrepancies may be attributed to the smaller sample sizes (*n* = 25) in the stratified subgroups and the exploratory nature of the *post hoc* analysis. Thus, insufficient statistical power precludes support for a definitive subgroup effect. These findings are underpowered and should be treated as hypothesis-generating. However, regarding the stratification by AHI, it is important to note that participants demonstrated a range of adherence levels and the AHI values only reflect baseline characteristics rather than the overall severity of OSA throughout the study period. Caution is warranted when interpreting these results.

The endpoint of this study represents an exploratory endpoint of the phase 3 trial in China, rather than a primary endpoint, and the isolated differences were not corrected for multiplicity, these findings should be interpreted with caution as they may represent false positives. Therefore, these results are hypothesis-generating, requiring validation through a pre-designed trial in the future. Additionally, CPAP adherence data was collected from self-report diaries rather than being objectively verified, there is a possibility of misclassification, which could bias subgroup analyses and attenuate potential treatment related signals. This study did not evaluate subgroups on the basis of CPAP use. A related limitation of our study is that changes in sleep quality on the second and third PSG assessments could be influenced by the removal of the first night effect in the sleep laboratory. In addition, given the high proportion of male participants (> 90%), the external validity of the current study findings for female participants with OSA-EDS is limited. Furthermore, given the exploratory nature of this analysis, the commonly used subjective scale tools such as the Pittsburgh Sleep Quality Index (PSQI) (43), were not included; instead, PSG was conducted to provide objective and reliable measurements of nighttime sleep quality. Another limitation of this study is its relatively short duration of 12 weeks. Although this timeframe provides valuable insights into the short-term effects of solriamfetol on nocturnal sleep quality and daytime wakefulness, it may not address the long-term effects.

## 5 Conclusion

In conclusion, solriamfetol administered upon awakening effectively manages EDS in patients with OSA without affecting nocturnal sleep quality. During treatment, no clinically significant or persistent changes were observed in PSG parameters, including TST, WASO, AI, and AHI, compared with the baseline or placebo. These findings were similar to those observed for other WPAs used in managing OSA-EDS. Overall, these results underscore the role of morning administration of solriamfetol as a reliable therapeutic option for improving wakefulness without compromising on sleep quality.

## Data Availability

The datasets presented in this article are not readily available because they are derived from a sponsor-owned Phase 3 clinical trial. Requests to access the datasets should be directed to the corresponding author YD (ndefy02029@ncu.edu.cn).

## Footnotes

1
www.chinadrugtrials.org.cn
