# Supplementary material for: Impact of solriamfetol treatment on sleep quality in Chinese patients with OSA-EDS: results of a randomized controlled trial
Source: Front Med (Lausanne). 2026 Feb 18;13:1712097. doi: 10.3389/fmed.2026.1712097 (PMC12958185; doi:10.3389/fmed.2026.1712097)
Supplement: Supplementary file 1 [file Data_Sheet_1.docx]

***Supplementary material***

**Impact of Solriamfetol Treatment on Sleep Quality in Chinese Patients with OSA-EDS —Results of a Randomized Controlled Trial**

**Sensitivity Analyses**

**Analysis of sleep quality-related changes from baseline – stratified by stability of adherence to primary OSA therapy** (**Supplementary Table 1**).

**Analysis of respiratory-related changes from baseline – stratified by stability of adherence to primary OSA therapy** (**Supplementary Table 2**).

**Demographics and Baseline Results – stratified by baseline AHI** (**Supplementary Table 3**).

**Sensitivity analysis of PSG parameters changes from baseline to Week 12 - based on baseline AHI group** (**Supplementary Table 4**).

**Supplementary Table 1 Analysis of sleep quality-related changes from baseline – stratified by stability of adherence to primary OSA therapy**

| **Variables** | **Placebo** | **Solriamfetol** | **Least Squares Mean Difference (95%CI) for Between-Group Comparison vs. Placebo** | **Intergroup *P* value vs Placebo** |
| --- | --- | --- | --- | --- |
| **TST (minutes)** |  |  |  |  |
| **Baseline** | **N=86** | **N = 85** |  |  |
| Mean (±SD) | 405.96 (40.537) | 400.61 (43.237) |  |  |
| Median (Q1, Q3) | 409.00 (386.50, 436.50) | 407.00 (367.50, 439.00) |  |  |
| **Week 2 change** | **N= 82** | **N=77** | 2.48 (-12.04, 17.00) | 0.7362 |
| Mean (±SD) | -6.33 (44.587) | -3.55 (51.202) |  |  |
| Median (Q1, Q3) | -0.50 (-20.00, 19.50) | -1.50 (-21.00, 18.00) |  |  |
| **Week 5** **change** | **N=83** | **N=81** | -4.57 (-18.88, 9.74) | 0.5292 |
| Mean (±SD) | 2.75 (44.713) | 0.08 (49.020) |  |  |
| Median (Q1, Q3) | 7.50 (-17.00, 30.50) | 6.00 (-24.00, 25.00) |  |  |
| **Week 12** **change** | **N=81** | **N=78** | -0.34 (-12.53, 11.85) | 0.9565 |
| Mean (±SD) | 5.59 (43.819) | 7.10 (38.285) |  |  |
| Median (Q1, Q3) | 9.50 (-16.00, 27.00) | 3.75 (-17.50, 30.50) |  |  |
| **Overall treatment effect P value** |  |  |  |  |
| Group |  | 0.8733 |  |  |
| Time |  | 0.0479 |  |  |
| Group*Time |  | 0.7048 |  |  |
| **N1 sleep time (minutes)** |  |  |  |  |
| **Baseline** | **N=85** | **N=85** |  |  |
| Mean (±SD) | 48.75 (31.052) | 55.78 (31.933) |  |  |
| Median (Q1, Q3) | 44.00 (27.00, 57.00) | 46.50 (35.50, 67.50) |  |  |
| **Week 2 change** | **N=82** | **N=77** | 9.35 (2.88, 15.81) | 0.0049 |
| Mean (±SD) | -6.90 (25.050) | -0.42 (21.981) |  |  |
| Median (Q1, Q3) | -7.25 (-15.00, 7.00) | 0.50 (-14.50, 15.00) |  |  |
| **Week 5** **change** | **N= 83** | **N=81** | 6.72 (0.25, 13.18) | 0.0418 |
| Mean (±SD) | -5.55 (23.620) | -1.99 (23.119) |  |  |
| Median (Q1, Q3) | -3.50 (-15.00, 6.50) | -0.50 (-15.00, 10.50) |  |  |
| **Week 12** **change** | **N=81** | **N=78** | 6.70 (-1.41, 14.81) | 0.1047 |
| Mean (SD) | -5.11 (28.071) | -1.81 (30.548) |  |  |
| Median (Q1, Q3) | -2.00 (-17.00, 10.50) | -4.75 (-19.00, 8.00) |  |  |
| **Overall treatment effect P value** |  |  |  |  |
| Group |  | 0.0100 |  |  |
| Time |  | 0.9801 |  |  |
| Group*Time |  | 0.6389 |  |  |
| **N2 sleep time (minutes)** |  |  |  |  |
| **Baseline** | **N=85** | **N=85** |  |  |
| Mean (±SD) | 231.35 (42.184) | 236.76 (44.445) |  |  |
| Median (Q1, Q3) | 235.50 (205.50, 259.00) | 238.00 (216.50, 264.00) |  |  |
| **Week 2 change** | **N=82** | **N=77** | -1.46 (-14.14, 11.21) | 0.8197 |
| Mean (SD) | 1.57 (43.381) | -4.69 (48.317) |  |  |
| Median (Q1, Q3) | -4.75 (-24.00, 32.00) | -10.50 (-28.00, 33.50) |  |  |
| **Week 5** **change** | **N=83** | **N=81** | 3.84 (-8.63, 16.30) | 0.5442 |
| Mean (±SD) | 2.03 (43.577) | 2.60 (47.136) |  |  |
| Median (Q1, Q3) | 2.00 (-28.50, 27.50) | 1.00 (-23.50, 36.50) |  |  |
| **Week 12** **change** | **N=81** | **N=78** | 2.90 (-10.36, 16.15) | 0.6667 |
| Mean (±SD) | 6.04 (47.660) | 5.94 (47.730) |  |  |
| Median (Q1, Q3) | 3.50 (-24.00, 34.00) | 8.50 (-22.00, 26.50) |  |  |
| **Overall treatment effect P value** |  |  |  |  |
| Group |  | 0.7335 |  |  |
| Time |  | 0.1242 |  |  |
| Group*Time |  | 0.7277 |  |  |
| **N3 sleep time** |  |  |  |  |
| **Baseline** | **N=85** | **N=85** |  |  |
| Mean (±SD) | 37.34 (33.047) | 33.20 (31.163) |  |  |
| Median (Q1, Q3) | 29.00 (10.00, 58.50) | 27.50 (4.00, 55.50) |  |  |
| **Week 2 change** | **N=82** | **N=77** | -0.30 (-7.18, 6.59) | 0.9324 |
| Mean (SD) | 0.85 (23.837) | 2.03 (22.048) |  |  |
| Median (Q1, Q3) | 0.75 (-15.50, 16.00) | 0.50 (-4.50, 15.50) |  |  |
| **Week 5** **change** | **N=83** | **N=81** | -7.85 (-14.69, -1.00) | 0.0251 |
| Mean (SD) | 3.48 (23.952) | -2.97 (22.409) |  |  |
| Median (Q1, Q3) | 1.50 (-11.00, 18.00) | 0.00 (-12.50, 9.50) |  |  |
| **Week 12** **change** | **N=81** | **N=78** | -3.00 (-9.76, 3.77) | 0.3830 |
| Mean (SD) | 1.18 (23.935) | -0.21 (21.160) |  |  |
| Median (Q1, Q3) | 2.00 (-14.50, 15.50) | 0.50 (-9.50, 12.00) |  |  |
| **Overall treatment effect P value** |  |  |  |  |
| Group |  | 0.1802 |  |  |
| Time |  | 0.7523 |  |  |
| Group*Time |  | 0.1214 |  |  |
| **WASO (minutes)** |  |  |  |  |
| **Baseline** | **N=85** | **N=85** |  |  |
| Mean (±SD) | 60.08 (36.361) | 66.55 (41.072) |  |  |
| Median (Q1, Q3) | 53.50 (32.00, 82.50) | 63.00 (28.50, 95.00) |  |  |
| **Week 2 change** | **N=82** | **N=77** | -6.31 (-18.35, 5.73) | 0.3019 |
| Mean (±SD) | 9.08 (38.226) | 2.17 (42.329) |  |  |
| Median (Q1, Q3) | 5.75 (-13.00, 24.00) | 1.00 (-16.00, 18.50) |  |  |
| **Week 5 change** | **N=83** | **N=81** | 0.34 (-10.52, 11.20) | 0.9502 |
| Mean (±SD) | -2.36 (34.704) | -4.38 (38.623) |  |  |
| Median (Q1, Q3) | -2.50 (-20.00, 15.50) | -6.50 (-26.00, 13.50) |  |  |
| **Week 12 change** | **N=81** | **N=78** | -4.62 (-15.22, 5.98) | 0.3904 |
| Mean (±SD) | -1.09 (37.405) | -7.41 (34.610) |  |  |
| Median (Q1, Q3) | -5.50 (-24.00, 18.50) | -5.50 (-30.00, 15.00) |  |  |
| **Overall treatment effect P value** |  |  |  |  |
| Group |  | 0.4029 |  |  |
| Time |  | 0.0088 |  |  |
| Group*Time |  | 0.5508 |  |  |

**Notes:** N = number of study participants in each group in the corresponding analysis set; Number of cases = number of study participants with non-missing parameter values in the corresponding group.

SD, standard deviation; TST, total sleep time; WASO, wakefulness after sleep onset; Q1, Q3, first and third quartile.

**Supplementary Table 2 Analysis of respiratory-related changes from baseline – stratified by stability of adherence to primary OSA therapy**

| **Variables** | **Placebo** | **Solriamfetol** | **Least Squares Mean Difference (95%CI) for Between-Group Comparison vs. Placebo** | **Intergroup *P* value vs Placebo** |
| --- | --- | --- | --- | --- |
| **Mean oxygen saturation , n (%)** |  |  |  |  |
| **Baseline** | **N=85** | **N = 85** |  |  |
| Mean (±SD) | 95.00 (1.890) | 95.18 (1.746) |  |  |
| Median (Q1, Q3) | 95.30 (94.10, 96.30) | 95.60 (94.60, 96.40) |  |  |
| **Week 2 change** | **N= 82** | **N=77** | -0.35 (-0.73, 0.04) | 0.0799 |
| Mean (±SD) | 0.17 (1.291) | -0.17 (1.237) |  |  |
| Median (Q1, Q3) | 0.15 (-0.40, 0.90) | -0.20 (-0.60, 0.60) |  |  |
| **Week 5 change** | **N=83** | **N=81** | -0.46 (-0.91, -0.00) | 0.0502 |
| Mean (±SD) | 0.13 (1.365) | -0.39 (1.726) |  |  |
| Median (Q1, Q3) | 0.00 (-0.50, 0.60) | 0.00 -0.70, 0.40 |  |  |
| **Week 12 change** | **N=81** | **N=78** | -0.46 (-0.90, -0.02) | 0.0427 |
| Mean (±SD) | 0.03 (1.438) | -0.44 (1.528) |  |  |
| Median (Q1, Q3) | -0.10 (-0.60, 0.60) | -0.20 (-0.90, 0.50) |  |  |
| **Overall treatment effect P value** |  |  |  |  |
| Group |  | 0.0183 |  |  |
| Time |  | 0.1555 |  |  |
| Group*Time |  | 0.8361 |  |  |
| **Mini SaO2 (%)** |  |  |  |  |
| **Baseline** | **N=85** | **N=85** |  |  |
| Mean (±SD) | 83.29 (10.822) | 84.66 (7.588) |  |  |
| Median (Q1, Q3) | 87.00 (78.00, 91.00) | 86.00 (81.00, 90.00) |  |  |
| **Week 2 change** | **N=82** | **N=77** | -0.54 (-2.18, 1.09) | 0.5115 |
| Mean (±SD) | 1.59 (8.049) | 0.47 (4.838) |  |  |
| Median (Q1, Q3) | 0.50 (-1.00, 3.00) | 0.00 (-1.00, 3.00) |  |  |
| **Week 5 change** | **N= 83** | **N=81** | -0.45 (-2.09, 1.18) | 0.4829 |
| Mean (±SD) | 1.17 (6.188) | -0.14 (5.882) |  |  |
| Median (Q1, Q3) | 0.00 (-2.00, 2.20) | 0.00 (-3.00, 4.00) |  |  |
| **Week 12 change** | **N=81** | **N=78** | -0.75 (-2.59, 1.10) | 0.4252 |
| Mean (±SD) | 0.72 (8.124) | -0.73 (5.488) |  |  |
| Median (Q1, Q3) | 0.00 (-3.00, 3.00) | -1.00 (-3.00, 2.00) |  |  |
| **Overall treatment effect P value** |  |  |  |  |
| Group |  | 0.4032 |  |  |
| Time |  | 0.0835 |  |  |
| Group*Time |  | 0.9545 |  |  |
| **AI (times/hour)** |  |  |  |  |
| **Baseline** | **N=85** | **N=85** |  |  |
| Mean (±SD) | 7.26 (13.013) | 8.75 (16.060) |  |  |
| Median (Q1, Q3) | 1.30 (0.20, 8.50) | 1.60 (0.30, 8.80) |  |  |
| **Week 2 change** | **N=82** | **N=77** | 1.27 (-1.57, 4.10) | 0.3788 |
| Mean (±SD) | -1.47 (13.543) | -0.83 (8.372) |  |  |
| Median (Q1, Q3) | -0.15 (-2.60, 0.40) | 0.00 (-0.90, 0.70) |  |  |
| **Week 5 change** | **N=83** | **N=81** | 2.08 (-0.68, 4.85) | 0.1383 |
| Mean (±SD) | -1.23 (9.746) | 0.20 (10.525) |  |  |
| Median (Q1, Q3) | 0.00 (-1.20, 0.80) | 0.00 (-1.30, 1.20) |  |  |
| **Week 12 change** | **N=81** | **N=78** | 1.95 (-1.27, 5.18) | 0.2335 |
| Mean (±SD) | -0.04 (10.455) | 1.16 (12.534) |  |  |
| Median (Q1, Q3) | 0.00 (-1.50, 1.50) | 0.00 (-1.10, 1.50) |  |  |
| **Overall treatment effect P value** |  |  |  |  |
| Group |  | 0.1105 |  |  |
| Time |  | 0.1431 |  |  |
| Group*Time |  | 0.8372 |  |  |
| **AHI (times/hour)** |  |  |  |  |
| **Baseline** | **N=85** | **N=85** |  |  |
| Mean (±SD) | 18.87 (21.833) | 19.67 (23.375) |  |  |
| Median (Q1, Q3) | 8.90 (2.30, 28.90) | 7.40 (1.60, 29.10) |  |  |
| **Week 2 change** | **N=82** | **N=77** | -1.24 (-5.54, 3.07) | 0.5715 |
| Mean (±SD) | 1.01 (16.705) | -0.47 (10.393) |  |  |
| Median (Q1, Q3) | -1.05 (-5.60, 1.60) | -0.10 (-2.90, 1.50) |  |  |
| **Week 5 change** | **N=83** | **N=81** | 2.37 (-0.75, 5.49) | 0.1357 |
| Mean (±SD) | -2.06 (10.803) | 0.29 (10.242) |  |  |
| Median (Q1, Q3) | -0.60 (-5.40, 2.20) | 0.00 (-3.40, 3.00) |  |  |
| **Week 12 change** | **N=81** | **N=78** | 2.77 (-0.55, 6.08) | 0.1010 |
| Mean (±SD) | -1.35 (10.210) | 0.94 (11.486) |  |  |
| Median (Q1, Q3) | -0.30 (-4.50, 2.10) | -0.05 (-2.90, 2.30) |  |  |
| **Overall treatment effect P value** |  |  |  |  |
| Group |  | 0.3483 |  |  |
| Time |  | 0.7106 |  |  |
| Group*Time |  | 0.1648 |  |  |
| **Number of apneas (times)** |  |  |  |  |
| **Baseline** | **N=85** | **N=85** |  |  |
| Mean (±SD) | 7.42 (16.620) | 4.87 (20.464) |  |  |
| Median (Q1, Q3) | 1.00 (0.00, 7.00) | 1.00 (0.00, 3.00) |  |  |
| **Week 2 change** | **N=82** | **N=77** | NA | 0.8474 |
| Mean (±SD) | -2.40 (16.651) | -1.92 (18.919) |  |  |
| Median (Q1, Q3) | 0.00 (-3.00, 2.00) | 0.00 (-1.00, 1.00) |  |  |
| **Week 5 change** | **N=83** | **N=81** | NA | 0.4911 |
| Mean (±SD) | -1.46 (12.264) | -0.84 (19.626) |  |  |
| Median (Q1, Q3) | 0.00 (-2.00, 1.00) | 0.00 (-1.00, 1.00) |  |  |
| **Week 12 change** | **N=81** | **N=78** | NA | 0.7653 |
| Mean (±SD) | -2.69 (13.825) | 0.23 (28.817) |  |  |
| Median (Q1, Q3) | 0.00 (-3.00, 1.00) | 0.00 (-1.00, 1.00) |  |  |

**Notes:** N = number of study participants in each group in the corresponding analysis set; Number of cases = number of study participants with non-missing parameter values in the corresponding group.

SD, standard deviation; AHI, apnea-hypopnea index; AI, apnea index; Mini SaO2, minimum oxygen saturation; Q1, Q3, first and third quartile.

**Supplementary Table 3 Demographics and Baseline Results – stratified by baseline AHI**

|  | | | | | | | | |
| --- | --- | --- | --- | --- | --- | --- | --- | --- |
|  | **Placebo** | | | | **Solriamfetol** | | | |
|  | AHI<5 (N=42) | 5≤AHI<15 (N=17) | 15≤AHI<30 (N=17) | AHI≥30 (N=23) | AHI<5 (N=43) | 5≤AHI<15 (N=20) | 15≤AHI<30 (N=15) | AHI≥30 (N=23) |
| **Age, years** |  |  |  |  |  |  |  |  |
| Number | 42 | 17 | 17 | 23 | 43 | 20 | 15 | 23 |
| Mean (±SD) | 45.5 (10.84) | 46.5 (14.71) | 44.1 (15.10) | 45.3 (12.89) | 45.8 (12.38) | 43.3 (11.89) | 48.4 (10.93) | 46.6 (11.59) |
| Median (Q1, Q3) | 47.0 (38.0, 52.0) | 49.0 (35.0, 60.0) | 40.0 (32.0, 55.0) | 40.0 (34.0, 55.0) | 47.0 (37.0, 50.0) | 41.0 (35.5, 50.5) | 50.0 (39.0, 59.0) | 46.0 (35.0, 58.0) |
| **Gender, n(%)** |  |  |  |  |  |  |  |  |
| Male | 37 (88.1) | 16 (94.1) | 14 (82.4) | 21 (91.3) | 40 (93.0) | 19 (95.0) | 12 (80.0) | 22 (95.7) |
| Female | 5 (11.9) | 1 (5.9) | 3 (17.6) | 2 (8.7) | 3 (7.0) | 1 (5.0) | 3 (20.0) | 1 (4.3) |
| **BMI (kg/m^2^)** |  |  |  |  |  |  |  |  |
| Number | 42 | 17 | 17 | 23 | 43 | 20 | 15 | 23 |
| Mean (±SD) | 27.15 (3.811) | 28.04 (4.150) | 27.61 (4.376) | 26.53 (3.405) | 28.40 (3.652) | 26.46 (3.035) | 28.45 (3.756) | 27.17 (2.614) |
| Median (Q1, Q3) | 26.80 (24.70, 29.60) | 27.30 (25.20, 31.50) | 26.90 (24.90, 30.40) | 24.90 (24.40, 28.00) | 27.90 (26.10, 30.40) | 26.70 (24.45, 29.20) | 28.20 (25.00, 30.50) | 27.10 (25.10, 29.40) |
| **Stratification factor** |  |  |  |  |  |  |  |  |
| Adherent to primary therapy | 33 (78.6) | 8 (47.1) | 6 (35.3) | 4 (17.4) | 35 (81.4) | 13 (65.0) | 2 (13.3) | 2 (8.7) |
| Non-adherent to primary therapy | 5 (11.9) | 7 (41.2) | 4 (23.5) | 3 (13.0) | 7 (16.3) | 3 (15.0) | 6 (40.0) | 4 (17.4) |
| No primary therapy | 4 (9.5) | 2 (11.8) | 7 (41.2) | 16 (69.6) | 1 (2.3) | 4 (20.0) | 7 (46.7) | 17 (73.9) |

**Notes**: N = number of study participants in each group in the corresponding analysis population; n (%) = number and percentage of study participants meeting a specific category, percentages will be calculated based on the number of study participants in each group that is not missing; number of cases = number of non-missing study participants in a specific category in the corresponding group.

**Abbreviations:** BMI, body mass index; SD, standard deviation; AHI, apnea-hypopnea index; Q1,Q3, first and third quartile

**Supplementary Table 4 Sensitivity analysis of PSG parameters changes from baseline to Week 12 - based on baseline AHI group**

|  | Placebo | | | | Solriamfetol | | | |
| --- | --- | --- | --- | --- | --- | --- | --- | --- |
| Variables | AHI<5 (N=42) | 5≤AHI<15 (N=17) | 15≤AHI<30 (N=17) | AHI≥30 (N=23) | AHI<5 (N=43) | 5≤AHI<15 (N=20) | 15≤AHI<30 (N=15) | AHI≥30 (N=23) |
| **TST (minutes)** | | | | | | | | |
| **Baseline** |  |  |  |  |  |  |  |  |
| Number | 42 | 17 | 17 | 23 | 43 | 20 | 15 | 23 |
| Mean (±SD) | 410.60 (34.424) | 402.76 (42.008) | 407.50 (47.868) | 399.37 (42.789) | 411.22 (36.084) | 399.03 (43.818) | 380.23 (41.667) | 404.41 (49.948) |
| Median (Q1, Q3) | 410.25 (391.00, 441.50) | 410.50 (379.00, 433.00) | 421.50 (363.00, 444.00) | 407.00 (378.50, 430.50) | 420.00 (382.50, 439.50) | 396.50 (366.75, 442.00) | 372.50 (340.50, 416.00) | 416.50 (367.50, 447.00) |
| Week 12 (changed from baseline) | | | | | | | | |
| Number | 37 | 17 | 14 | 22 | 41 | 18 | 13 | 20 |
| Mean (±SD) | -0.57 (30.060) | 13.62 (34.140) | 19.14 (58.131) | 4.23 (55.702) | -0.11 (35.763) | 14.17 (50.639) | 15.19 (36.596) | 7.65 (30.392) |
| Median (Q1, Q3) | 2.50 (-20.50, 13.00) | 20.00 (-4.50, 30.00) | 14.75 (8.50, 36.00) | 4.00 (-29.00, 49.00) | 0.00 (-20.00, 12.50) | 21.25 (-24.50, 61.50) | 28.50 (-17.00, 38.00) | 1.75 (-9.50, 32.50) |
| Least Squares Mean Difference (95%CI) vs Placebo |  |  |  |  | -0.08 (-13.91, 13.76) | -1.43 (-30.69, 27.84) | -6.58 (-44.62, 31.47) | 2.61 (-24.27, 29.49) |
| P value |  |  |  |  | 0.9911 | 0.9216 | 0.7256 | 0.8452 |
| **N1 sleep time (minutes)** | | | | | | | | |
| **Baseline** |  |  |  |  |  |  |  |  |
| Number | 42 | 17 | 17 | 23 | 43 | 20 | 15 | 23 |
| Mean (±SD) | 40.87 (26.415) | 35.06 (16.366) | 56.24 (37.384) | 62.41 (30.712) | 37.20 (15.076) | 43.68 (22.672) | 63.23 (21.320) | 89.26 (47.214) |
| Median (Q1, Q3) | 34.50 (23.00, 50.00) | 38.50 (23.50, 44.00) | 45.00 (32.50, 70.00) | 54.00 (45.50, 74.00) | 36.50 (27.50, 46.00) | 39.75 (25.75, 56.75) | 59.50 (47.00, 76.50) | 96.00 (52.00, 112.00) |
| Week 12 (changed from baseline) | | | | | | | | |
| Number | 37 | 17 | 14 | 22 | 41 | 18 | 13 | 20 |
| Mean (±SD) | -3.46 (24.444) | -0.97 (16.047) | -22.14 (34.835) | -0.84 (30.711) | 2.26 (18.965) | -1.19 (23.700) | -19.65 (20.453) | 0.03 (49.689) |
| Median (Q1, Q3) | 2.50 (-8.50, 13.50) | -4.00 (-12.00, 12.00) | -19.00 (-29.50, 2.00) | -2.50 (-17.50, 13.00) | 1.50 (-10.00, 10.00) | -4.50 (-18.00, 11.00) | -19.50 (-22.00, -6.00) | -7.25 (-34.00, 22.00) |
| Least Squares Mean Difference (95%CI) vs Placebo |  |  |  |  | 4.69 (-3.32, 12.70) | 4.41 (-6.81, 15.62) | 8.57 (-4.70, 21.84) | 8.71 (-15.40, 32.82) |
| P value |  |  |  |  | 0.2469 | 0.4302 | 0.1963 | 0.4704 |
| **N2 sleep time (minutes)** | | | | | | | | |
| **Baseline** |  |  |  |  |  |  |  |  |
| Number | 42 | 17 | 17 | 23 | 43 | 20 | 15 | 23 |
| Mean (±SD) | 243.49 (34.107) | 230.18 (34.383) | 214.32 (52.314) | 232.09 (47.919) | 249.02 (39.053) | 228.28 (40.133) | 228.67 (39.479) | 227.41 (59.190) |
| Median (Q1, Q3) | 245.00 (220.00, 261.00) | 234.50 (205.50, 254.00) | 214.50 (187.00, 248.50) | 233.00 (193.00, 270.00) | 258.00 (227.00, 266.50) | 231.50 (203.50, 248.25) | 235.50 (205.50, 254.50) | 226.50 (166.50, 247.50) |
| Week 12 (changed from baseline) | | | | | | | | |
| Number | 37 | 17 | 14 | 22 | 41 | 18 | 13 | 20 |
| Mean (±SD) | -7.89 (46.240) | 7.97 (41.611) | 33.54 (55.147) | 7.59 (53.166) | -1.84 (44.551) | 13.78 (47.981) | 7.15 (47.798) | 5.50 (53.961) |
| Median (Q1, Q3) | 0.50 (-31.00, 21.50) | -3.50 (-12.00, 30.00) | 29.75 (9.00, 53.50) | -0.25 (-31.50, 34.00) | 7.50 (-29.00, 21.00) | 15.75 (-17.00, 29.00) | 6.00 (-16.50, 31.50) | 3.75 (-22.00, 42.75) |
| Least Squares Mean Difference (95%CI) vs Placebo |  |  |  |  | 11.60 (-6.25, 29.45) | 6.49 (-19.47, 32.44) | -21.12 (-57.97, 15.74) | -4.79 (-35.62, 26.05) |
| P value |  |  |  |  | 0.1994 | 0.6148 | 0.2501 | 0.7556 |
| **N3 sleep time (minutes)** | | | | | | | | |
| **Baseline** |  |  |  |  |  |  |  |  |
| Number | 42 | 17 | 17 | 23 | 43 | 20 | 15 | 23 |
| Mean (±SD) | 36.13 (25.845) | 40.53 (26.415) | 46.59 (52.595) | 28.02 (25.189) | 37.03 (31.118) | 48.08 (31.040) | 26.27 (25.186) | 23.02 (30.563) |
| Median (Q1, Q3) | 31.50 (16.50, 50.50) | 29.50 (25.50, 60.50) | 29.50 (3.50, 66.50) | 24.00 (6.50, 42.50) | 38.00 (6.50, 55.50) | 49.25 (20.00, 72.00) | 26.50 (0.50, 41.50) | 4.50 (0.00, 56.00) |
| **Week 12 (changed from baseline)** | | | | | | | | |
| Number | 37 | 17 | 14 | 22 | 41 | 18 | 13 | 20 |
| Mean (±SD) | 8.99 (34.350) | 6.82 (20.315) | -1.82 (20.939) | -8.59 (25.338) | -2.85 (22.203) | -2.69 (27.379) | 8.04 (15.311) | 4.85 (26.559) |
| Median (Q1, Q3) | 5.00 (-7.00, 27.00) | 5.50 (-1.50, 19.50) | -2.50 (-16.50, 8.50) | -5.75 (-27.00, 9.00) | 1.00 (-20.50, 7.50) | 1.25 (-14.00, 15.00) | 3.50 (-5.00, 19.00) | 0.00 (-7.75, 11.50) |
| Least Squares Mean Difference (95%CI) vs Placebo |  |  |  |  | -13.57 (-25.49, -1.64) | -7.54 (-22.51, 7.43) | 8.77 (-6.48, 24.02) | 11.87 (-3.31, 27.06) |
| P value |  |  |  |  | 0.0263 | 0.3130 | 0.2477 | 0.1220 |
| **WASO (minutes)** | | | | | | | | |
| **Baseline** |  |  |  |  |  |  |  |  |
| Number | 42 | 17 | 17 | 23 | 43 | 20 | 15 | 23 |
| Mean (±SD) | 56.48 (31.272) | 60.74 (38.776) | 56.71 (40.704) | 68.54 (38.320) | 57.40 (36.342) | 69.25 (44.749) | 85.00 (36.929) | 61.35 (44.547) |
| Median (Q1, Q3) | 48.75 (32.00, 74.00) | 64.00 (25.00, 83.50) | 51.50 (32.50, 68.50) | 63.00 (47.00, 89.00) | 44.50 (28.00, 86.00) | 74.50 (19.00, 98.75) | 91.00 (51.00, 112.00) | 55.00 (21.50, 98.50) |
| **Week 12 (changed from baseline)** | | | | | | | | |
| Number | 37 | 17 | 14 | 22 | 41 | 18 | 13 | 20 |
| Mean (±SD) | 2.59 (28.150) | -3.94 (33.387) | -11.04 (42.500) | 0.39 (46.625) | 1.57 (30.067) | -15.50 (50.779) | -21.96 (32.247) | -3.63 (25.613) |
| Median (Q1, Q3) | -2.00 (-17.00, 23.50) | -11.00 (-25.50, 14.50) | -14.25 (-19.50, -1.00) | -0.75 (-27.50, 34.50) | -1.00 (-12.00, 19.00) | -18.50 (-60.00, 14.00) | -29.00 (-39.00, -6.00) | -1.00 (-19.75, 11.00) |
| Least Squares Mean Difference (95%CI) vs Placebo |  |  |  |  | -0.33 (-12.61, 11.95) | -7.52 (-35.43, 20.39) | -3.65 (-31.43, 24.13) | -5.75 (-28.98, 17.48) |
| P value |  |  |  |  | 0.9577 | 0.5874 | 0.7898 | 0.6200 |
| **AHI (times/hour)** | | | | | | | | |
| **Baseline** |  |  |  |  |  |  |  |  |
| Number | 42 | 17 | 17 | 23 | 43 | 20 | 15 | 23 |
| Mean (±SD) | 1.53 (1.270) | 8.69 (2.631) | 21.60 (3.908) | 50.49 (17.099) | 1.58 (1.327) | 8.51 (3.245) | 21.49 (5.023) | 57.08 (16.966) |
| Median (Q1, Q3) | 1.10 (0.40, 2.40) | 7.90 (7.10, 9.80) | 20.20 (18.80, 25.30) | 43.50 (38.90, 60.30) | 1.30 (0.60, 2.40) | 7.10 (5.75, 11.35) | 20.40 (16.70, 27.20) | 50.80 (44.20, 71.70) |
| **Week 12 (changed from baseline)** | | | | | | | | |
| Number | 37 | 17 | 14 | 22 | 41 | 18 | 13 | 20 |
| Mean (±SD) | 1.31 (4.124) | -2.79 (3.722) | -1.80 (13.513) | -4.50 (15.291) | 1.27 (6.069) | 3.56 (10.605) | -3.44 (8.947) | 0.54 (17.739) |
| Median (Q1, Q3) | 0.60 (-0.50, 1.80) | -4.10 (-5.30, 0.90) | -3.85 (-12.20, 5.90) | -2.80 (-13.80, 3.40) | 0.10 (-0.70, 0.70) | -1.20 (-3.00, 11.00) | -5.00 (-8.40, 0.20) | -0.50 (-9.25, 14.65) |
| Least Squares Mean Difference (95%CI) vs Placebo |  |  |  |  | - 0.00 (-2.36, 2.35) | 6.26 (0.67, 11.86) | -1.04 (-10.09, 8.02) | 7.02 (-3.13, 17.17) |
| P value |  |  |  |  | 0.9970 | 0.0294 | 0.8161 | 0.1701 |
| **AI (times/hour)** | | | | | | | | |
| **Baseline** |  |  |  |  |  |  |  |  |
| Number | 42 | 17 | 17 | 23 | 43 | 20 | 15 | 23 |
| Mean (±SD) | 0.33 (0.442) | 2.99 (3.369) | 5.26 (6.036) | 21.96 (17.542) | 0.35 (0.472) | 3.27 (3.078) | 7.55 (7.480) | 28.10 (24.207) |
| Median (Q1, Q3) | 0.10 (0.00, 0.40) | 1.60 (1.00, 3.90) | 2.30 (1.00, 9.50) | 19.80 (10.40, 27.70) | 0.30 (0.00, 0.40) | 1.90 (0.95, 5.05) | 5.10 (1.90, 13.60) | 25.40 (8.40, 45.30) |
| **Week 12 (changed from baseline)** | | | | | | | | |
| **Baseline** |  |  |  |  |  |  |  |  |
| Number | 37 | 17 | 14 | 22 | 41 | 18 | 13 | 20 |
| Mean (±SD) | 0.33 (1.472) | -1.48 (3.369) | 0.71 (7.546) | 1.26 (19.628) | 0.95 (5.876) | 2.50 (8.100) | -3.11 (10.896) | 2.80 (20.566) |
| Median (Q1, Q3) | 0.00 (-0.10, 0.40) | -1.30 (-2.40, -0.30) | -0.70 (-2.30, 1.60) | 4.20 (-15.10, 15.90) | 0.00 (-0.30, 0.20) | 0.00 (-1.20, 3.10) | -2.30 (-11.10, 2.10) | 1.50 (-10.85, 10.70) |
| Least Squares Mean Difference (95%CI) vs Placebo |  |  |  |  | 0.68 (-1.28, 2.64) | 4.03 (-0.38, 8.44) | -2.24 (-8.11, 3.62) | 5.38 (-5.30, 16.05) |
| P value |  |  |  |  | 0.4906 | 0.0722 | 0.4375 | 0.3153 |
| **Mean SaO2, n (%)** | | | | | | | | |
| **Baseline** |  |  |  |  |  |  |  |  |
| Number | 42 | 17 | 17 | 23 | 43 | 20 | 15 | 23 |
| Mean (±SD) | 95.46 (1.894) | 95.55 (1.544) | 95.42 (1.154) | 93.69 (1.843) | 95.89 (1.413) | 95.34 (1.650) | 94.67 (1.962) | 94.03 (1.624) |
| Median (Q1, Q3) | 96.00 (94.50, 96.90) | 95.60 (95.10, 96.50) | 95.50 (94.70, 96.20) | 93.50 (92.50, 95.30) | 96.20 (95.50, 96.70) | 95.55 (94.60, 96.35) | 95.40 (92.70, 96.30) | 94.60 (92.90, 95.10) |
| **Week 12 (changed from baseline)** | | | | | | | | |
| Number | 37 | 17 | 14 | 22 | 41 | 18 | 13 | 20 |
| Mean (±SD) | 0.00 (1.210) | 0.52 (1.662) | -0.31 (1.399) | -0.04 (1.431) | -0.21 (1.409) | -0.49 (1.232) | -0.29 (1.970) | -0.43 (1.624) |
| Median (Q1, Q3) | 0.00 (-0.30, 0.40) | 0.10 (-0.50, 0.70) | -0.40 (-0.90, 0.40) | -0.15 (-1.10, 0.80) | -0.10 (-0.60, 0.30) | -0.40 (-1.20, 0.40) | 0.40 (-0.60, 0.90) | -0.35 (-1.20, 0.80) |
| Least Squares Mean Difference (95%CI) vs Placebo |  |  |  |  | -0.15 (-0.71, 0.40) | -1.05 (-1.96, -0.14) | 0.02 (-1.32, 1.35) | -0.40 (-1.36, 0.56) |
| P value |  |  |  |  | 0.5894 | 0.0251 | 0.9801 | 0.4056 |
| **Mini SaO2 at baseline, n (%)** | | | | | | | | |
| **Baseline** |  |  |  |  |  |  |  |  |
| Number | 42 | 17 | 17 | 23 | 43 | 20 | 15 | 23 |
| Mean (±SD) | 91.12 (2.340) | 85.24 (10.109) | 80.65 (8.200) | 72.64 (10.342) | 89.33 (4.497) | 86.60 (4.500) | 83.60 (5.654) | 75.70 (8.694) |
| Median (Q1, Q3) | 92.00 (89.00, 93.00) | 88.00 (86.00, 90.00) | 83.00 (77.00, 87.00) | 75.00 (66.00, 81.00) | 90.00 (87.00, 92.00) | 87.50 (84.50, 90.00) | 83.00 (81.00, 88.00) | 78.00 (75.00, 81.00) |
| **Week 12 (changed from baseline)** | | | | | | | | |
| Number | 37 | 17 | 14 | 22 | 41 | 18 | 13 | 20 |
| Mean (±SD) | -1.11 (4.248) | 3.71 (11.789) | 1.07 (10.057) | 0.64 (6.616) | 0.29 (3.926) | -2.22 (4.894) | 1.62 (5.316) | -2.45 (7.178) |
| Median (Q1, Q3) | -1.00 (-2.00, 1.00) | 1.00 (-1.00, 3.00) | -1.50 (-4.00, 4.00) | 0.00 (-3.00, 4.00) | 0.00 (-2.00, 2.00) | -1.50 (-6.00, 1.00) | 2.00 (-2.00, 4.00) | -1.50 (-8.00, 3.50) |
| Least Squares Mean Difference (95%CI) vs Placebo |  |  |  |  | 0.34 (-1.45, 2.13) | -5.04 (-9.36, -0.73) | 4.00 (-1.12, 9.12) | -1.54 (-5.53, 2.46) |
| P value |  |  |  |  | 0.7060 | 0.0235 | 0.1207 | 0.4416 |
| **Number of apneas (times)** | | | | | | | | |
| **Baseline** |  |  |  |  |  |  |  |  |
| Number | 42 | 17 | 17 | 23 | 43 | 20 | 15 | 23 |
| Mean (±SD) | 1.55 (2.442) | 9.65 (13.233) | 7.94 (16.558) | 14.65 (26.913) | 1.21 (2.099) | 3.55 (3.486) | 2.87 (3.796) | 12.43 (38.601) |
| Median (Q1, Q3) | 0.00 (0.00, 2.00) | 3.00 (0.00, 17.00) | 1.00 (0.00, 6.00) | 2.00 (0.00, 26.00) | 0.00 (0.00, 2.00) | 2.50 (0.50, 6.50) | 2.00 (0.00, 3.00) | 1.00 (0.00, 8.00) |
| **Week 12 (changed from baseline)** | | | | | | | | |
| Number | 37 | 17 | 14 | 22 | 41 | 18 | 13 | 20 |
| Mean (±SD) | 0.84 (4.735) | -6.47 (11.086) | 2.14 (6.949) | -7.91 (22.179) | 0.61 (4.505) | 11.56 (40.140) | -0.31 (5.056) | -7.00 (44.588) |
| Median (Q1, Q3) | 0.00 (0.00, 2.00) | -1.00 (-12.00, 0.00) | 0.00 (-1.00, 2.00) | 0.00 (-8.00, 1.00) | 0.00 (-1.00, 0.00) | -0.50 (-2.00, 1.00) | -1.00 (-3.00, 0.00) | 0.00 (-2.00, 2.50) |
| Least Squares Mean Difference (95%CI) vs Placebo |  |  |  |  | NA | NA | NA | NA |
| P value |  |  |  |  | 0.3499 | 0.1261 | 0.3221 | 0.4897 |

**Abbreviations:** SD, standard deviation; TST, total sleep time; WASO, wakefulness after sleep onset; AHI, apnea-hypopnea index; AI, apnea index; Mini SaO2, minimum oxygen saturation; Q1, Q3, first and third quartile
